# Competing biomedical HIV prevention strategies: potential cost‐effectiveness of HIV vaccines and PrEP in Seattle, WA

**DOI:** 10.1002/jia2.25373

**Published:** 2019-08-11

**Authors:** Blythe Adamson, Louis Garrison, Ruanne V Barnabas, Josh J Carlson, James Kublin, Dobromir Dimitrov

**Affiliations:** ^1^ Department of Pharmacy The Comparative Health Outcomes, Policy, and Economics (CHOICE) Institute University of Washington Seattle WA USA; ^2^ Vaccine and Infectious Diseases Division Fred Hutchinson Cancer Research Center Seattle WA USA; ^3^ Flatiron Health New York NY USA; ^4^ Division of Allergy and Infectious Diseases Department of Global Health University of Washington Seattle WA USA; ^5^ HIV Vaccine Trials Network Fred Hutchinson Cancer Research Center Seattle WA USA

**Keywords:** HIV vaccines, pre‐exposure prophylaxis, cost‐benefit analysis, models, economic, costs and cost analysis, economic competition, disease transmission, infectious, preventive medicine

## Abstract

**Introduction:**

Promising HIV vaccine candidates are steadily progressing through the clinical trial pipeline. Once available, HIV vaccines will be an important complement but also potential competitor to other biomedical prevention tools such as pre‐exposure prophylaxis (PrEP). Accordingly, the value of HIV vaccines and the policies for rollout may depend on that interplay and tradeoffs with utilization of existing products. In this economic modelling analysis, we estimate the cost‐effectiveness of HIV vaccines considering their potential interaction with PrEP and condom use.

**Methods:**

We developed a dynamic model of HIV transmission among the men who have sex with men population (MSM), aged 15‐64 years, in Seattle, WA offered PrEP and HIV vaccine over a time horizon of 2025‐2045. A healthcare sector perspective with annual discount rate of 3% for costs (2017 USD) and quality‐adjusted life years (QALYs) was used. The primary economic endpoint is the incremental cost‐effectiveness ratio (ICER) when compared to no HIV vaccine availability.

**Results:**

HIV vaccines improved population health and increased healthcare costs. Vaccination campaigns achieving 90% coverage of high‐risk men and 60% coverage of other men within five years of introduction are projected to avoid 40% of new HIV infections between 2025 and 2045. This increased total healthcare costs by $30 million, with some PrEP costs shifted to HIV vaccine spending. HIV vaccines are estimated to have an ICER of $42,473/QALY, considered cost‐effective using a threshold of $150,000/QALY. Results were most sensitive to HIV vaccine efficacy and future changes in the cost of PrEP drugs. Sensitivity analysis found ranges of 30‐70% HIV vaccine efficacy remained cost‐effective. Results were also sensitive to reductions in condom use among PrEP and vaccine users.

**Conclusions:**

Access to an HIV vaccine is desirable as it could increase the overall effectiveness of combination HIV prevention efforts and improve population health. Planning for the rollout and scale‐up of HIV vaccines should carefully consider the design of policies that guide interactions between vaccine and PrEP utilization and potential competition.

## 1 Introduction

Experts say an HIV vaccine is necessary, but not sufficient to end HIV 1, 2. A 50% effective vaccine may be good enough, but not enough. There are many exciting biomedical HIV prevention candidates in the research and development pipeline. Combinations of evidence‐based HIV treatment and prevention interventions will be necessary for eradication. Investment and policy decisions consider not only effectiveness, but also aspects of access, acceptability, behaviour change and costs. This is an important question because current prevention interventions are imperfect. Decision‐makers weigh population‐level tradeoffs for opportunities that offer small benefits to a large number of individuals or large benefits to a small number of individuals. For an affordable public health programme, substantially reduced drug prices will likely be needed 3, 4.

In the United States, Seattle, Washington is a national leader and early adopter of novel evidence‐based HIV strategies. Seattle‐King County Public Health surveillance rigorously monitors epidemic indicators and care cascade milestones, and it was the first US urban city to reach the “90‐90‐90” goal set by WHO. In King County, health officials estimate 6980 residents lived with diagnosed HIV infection in 2014, totalling more than half of all HIV cases in the state 5. Approximately 50,000 men who have sex with men (MSM) live in King County. In 2014, 281 people were newly diagnosed with HIV, with local data suggesting rectal gonorrhoea or early syphilis as one of the strongest risk factors 6.

Seattle has combated new infections with pre‐exposure prophylaxis (PrEP). As a complement to national guidelines for prescribing and monitoring PrEP 7, the local Public Health Seattle & King County with the Washington State Department of Health guide medical providers to recommend and discuss PrEP with target populations 8. PrEP users are recommended to get tested every three months for HIV and other sexually transmitted infections (STIs); adherence and retention can be a challenge. One US study of patients prescribed PrEP at least six months beforehand (n = 171), 72% were retained in care at three months and 57% were retained in PrEP care at six months 9. Long‐acting injectable cabotegravir for PrEP is also under investigation. Future evaluation of novel PrEP products may deem placebo‐controlled trials unethical.

Recent progress in HIV vaccine development means another biomedical product for prevention is approaching the horizon of availability 10. A breakthrough 2009 Phase 3 trial in Thailand found significant HIV vaccine efficacy averaging 31% fewer infections over three years 11. Confirmatory trials are ongoing in South Africa, with modifications to improve the Thai regimen – powered to detect HIV vaccine efficacy of 50% 12. Based on prospectively defined immunogenicity thresholds, criteria were met for the Pox‐Protein Public Private Partnership (P5) to support the launch of a Phase 2b/3 study in Africa: HIV Vaccine Trials Network (HVTN) 702 Study 13.

Real tradeoffs have to be made when offering imperfect prevention products. This is an important question because all available biomedical products for HIV prevention are imperfect, and the evidence of PrEP cost‐effectiveness is mixed 4, 14, 15, 16. One framework for optimal resource allocation for investments is a static optimization model to evaluate potential combination HIV prevention strategies 17 Previous modelling studies have separately examined the cost‐effectiveness of PrEP 4, 15, 18 and the potential cost‐effectiveness of HIV vaccines 19, 20, 21, 22, 23, 24, 25, 26, 27, 28, 29. Two models to date have evaluated the expected combined impact of vaccine and PrEP, assuming independent coverage targets are achieved with each tool, and both found lower costs and improved health outcomes compared with PrEP alone 27, 30. This is the first modelling study to examine the economic impact of potential competition and interaction between HIV vaccine and PrEP considering the potential interaction between them.

## 2 Methods

We conducted a health economic modelling analysis to estimate the impact and cost‐effectiveness of an HIV vaccine offered alongside PrEP in Seattle, WA.

### 2.1 Study population

The study population includes MSM ages 15‐64 in Seattle, WA, using Public Health Seattle‐King County and the Washington State Department of Health reports as the primary source for population data 5, 31, 32. Costs and benefits are evaluated over the time horizon 2025‐2045. The Department of Health estimates MSM account for 5.4% of the population in this age range 5. Within this population, 80% self‐identify as gay and more than 31% had six or more male sex partners in the last 12 months 31.

### 2.2 Model overview

**Figure 1:**
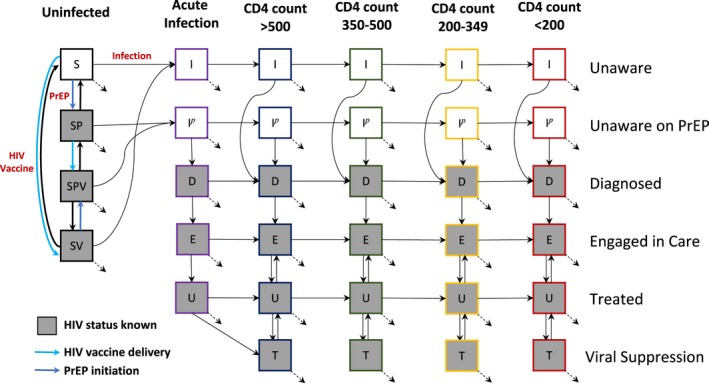
Schematic model diagram. This schematic represents the deterministic dynamic compartmental model. The boxes represent disease‐stage compartments of men who have sex with men and the arrows represent transitions between compartments. Individuals enter into the unvaccinated population and may die or exit the population at various disease stages. Not represented in the diagram is stratification by age group (15‐24, 24‐44 and 45‐64 years), risk group (low and high) and sexual role (anal insertive, receptive or versatile). PrEP, pre‐exposure prophylaxis.

We developed a deterministic dynamic compartmental mathematical model (Figure 1) to simulate the HIV epidemic among MSM in Seattle beginning in 2004. The model was already used to study the effectiveness of rapid antiretroviral therapy initiation among MSM in Peru 33. It consists of a system of differential equations describing HIV transmission and disease progression through a series of health states. Over time, MSM enter the population at age of sexual debut and exit the population at age 64. The population is stratified into groups by HIV infection status (susceptible and infected), age (<25, 25‐40, >40 years), risk of infection (low and high) and prevention modality (PrEP use and/or vaccination status). Infected MSM progress through a series of health states based on CD4‐count, treatment status and viral suppression (Appendix S1).

The model is used to simulate the HIV epidemic without an HIV vaccine to provide a reference scenario for the evaluation of the vaccine impact. PrEP is introduced in 2015 followed by HIV vaccine availability in 2025. The effectiveness of the intervention over the 2025‐2045 period is evaluated by comparison of the intervention to the reference scenario assuming no changes in the current Centers for Disease Control (CDC) guidelines for HIV prevention and treatment 7. Reported metrics represent the mean outcome of 100 simulations using the preselected sets of epidemic parameters identified in the calibration procedure described below.

We followed recommendations from the ISPOR‐SMDM Dynamic Transmission Modeling Task Force and the Second Panel on Cost‐Effectiveness in Health and Medicine 34, 35, 36. The model was developed in C++ and R version 3.4.2 37.

### 2.3 Model parameterization and calibration

The model is parameterized with epidemiological data representative of the HIV epidemic among MSM in Seattle. Calibration occurs from 2004 to 2014. Demographic and sexual behaviour characteristics including average number of partners per year, frequency of sex acts, proportion of acts protected by condoms, and lifetime duration of sexual activity were collected from published data (Tables 1, S2 and S3). Parameterization of age and risk group sexual mixing patters were imputed from a meta‐analyses of MSM that included a sample from Seattle 38. King County HIV prevalence was calibrated to the 2015 HIV/AIDS Epidemiology Report from the Public Health Seattle‐King County and Washington State Department of Health 5 and the CDC‐sponsored 2014 Seattle area National HIV Behavioral Survey of Men Who Have Sex with Men Sexual (NHBS‐MSM4) informed values for sexual risk behaviours, HIV testing and PrEP use 39. “High‐risk” was defined as MSM having five or more partners in the past 12 months, as a surrogate for the many risk factors identified in Seattle's clinical guidelines for PrEP use 7.

**Table 1:** Dynamic transmission model inputs

	Value	Source
Parameter		
Population size, men who have sex with men, ages 15‐64 years, King County, 2004	45,000	US Census 67
Fraction young, 15‐24 years	0.168	US Census Reporter 68
Fraction middle‐aged, 25‐44 years	0.463	US Census Reporter 68
Male maturation rate, rate of ageing into the population	0.03	Estimated
Fraction of high risk MSM of HIV infection (>6 partners in the last 12 months)		
Among young, 15‐24 years	0.310	Seattle HIV/AIDS Epi Report 31
Among middle‐aged, 25‐44 years	0.099	Seattle HIV/AIDS Epi Report 31
Among old, 45+ years	0.065	Seattle HIV/AIDS Epi Report 31
Insertive anal sex role, fraction of population with the role group “insertive”	0.325	Table 7, CDC 2016 69
Versatile anal sex role, fraction of population with the role group “versatile”	0.268	Table 7, CDC 2016 69
Number of sexual partners in the past 12 months		
High‐risk with young adults	10.5	Table 11, CDC 2016 69
High‐risk with middle‐aged	10.5	Table 11, CDC 2016 69
High‐risk with older adults	6.0	Table 11, CDC 2016 69
Low‐risk with young adults	1.5	Wall 2015 70
Low‐risk with middle‐aged	1.5	Wall 2015 70
Low‐risk with older adults	1.0	Wall 2015 70
Death rate, non‐AIDS, probability of dying between age x (midpoint of age category) and x + 1		
Ages 15‐24 years	0.001319	Life tables, Arias 2016 71
Ages 25‐44 years	0.001574	Life tables, Arias 2016 71
Ages 45‐64 years	0.008438	Life tables, Arias 2016 71
HIV vaccine efficacy	0.50	Expert opinion
HIV vaccine durability, average, years	5	Expert opinion
HIV prevention effectiveness		
Condom efficacy, reduction in susceptibility per act	0.7‐0.9	Smith 2015 72
Fraction of acts protected by a condom for		
Susceptible individuals, unvaccinated and not using PrEP	0.63	Seattle HIV/AIDS Epi Report 5
PrEP users	0.125	Montano 2017 32
Vaccinated, low‐risk	0.125	Assumed similar to PrEP users
Vaccinated, high‐risk	0.63	Assumed similar to susceptible low‐risk men
PrEP efficacy, reduction in susceptibility per act	0.80	Molina 2015, McCormack 2015 40, 41
Calibration targets		
HIV prevalence among MSM in King County	0.13‐0.17	Seattle HIV/AIDS Epi Report 31
Fraction of population who are diagnosed	0.72‐0.93	Seattle HIV/AIDS Epi Report 31
Fraction of diagnosed MSM who are engaged in care	0.88‐0.94	Seattle HIV/AIDS Epi Report 31
Fraction of infected MSM on ART who are virally suppressed	0.8‐0.86	Seattle HIV/AIDS Epi Report 31
Utilities		
Acute infection	0.69	Whitham 2016 73
CD4 count > 500 or viral suppression	0.73	Whitham 2016 73
CD4 count 350‐500	0.71	Whitham 2016 73
CD4 count 200‐349	0.69	Whitham 2016 73
CD4 count < 200	0.69	Whitham 2016 73
Costs, USD 2017		
Clinic visit for HIV prevention services, at each dose of HIV vaccine and/or each quarter of PrEP use		
Preventive medicine counselling, 30 minute office visit	51	National Physician Fee Schedule Relative Value File 74, HCPCS code 99402 (0.98 RVUs)
Laboratory tests, total	164	NASTAD PrEP Billing Code Guide 75 and CMS 2017 Clinical Diagnostic Laboratory Fee Schedule, mid‐point
HIV, fourth generation test	44	CPT^®^ code 87389
Chlamydia test	22	CPT^®^ code 86631
Gonorrhoea test	37	CPT^®^ code 87590
Syphilis test	25	CPT^®^ code 86780
Hepatitis B test	19	CPT^®^ code 87340
Measurement of blood urea and nitrogen serum creatinine levels	17	CPT^®^ codes 84520 and 82565
PrEP medication, 30‐day supply	1050	FSS price, 2017, US Veterans Affairs 60
HIV vaccine, cost per series	820	Expert opinion: assume 30% increasing benchmark compared to FSS price of GARDASIL‐9^®^ HPV vaccine 60
HIV care costs, quarterly		
CD4 count > 500	5872	Gebo 2010 77, Farnham 2013 78
CD4 count 350‐500	5959	Gebo 2010 77, Farnham 2013 78
CD4 count 200‐349	6915	Gebo 2010 77, Farnham 2013 78
CD4 count < 200	14,378	Gebo 2010 77, Farnham 2013 78

Costs have been adjusted to a common currency of 2017 USD.

ART, antiretroviral therapy; CDC, Centers for Disease Control; CMS, Centers for Medicare and Medicaid Services; FSS, Federal Supply Schedule; HPV, human papillomavirus; MSM, men who have sex with men; PrEP, pre‐exposure prophylaxis; RVUs, relative value units.

### 2.4 Interventions

#### 2.4.1 Pre‐exposure prophylaxis

**Figure 2:**
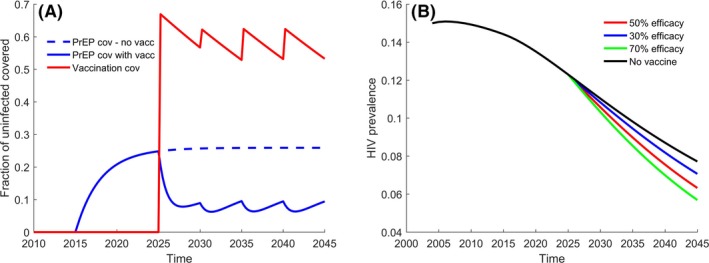
(A) Interactions in the utilization of PrEP with HIV vaccines and (B) projected prevalence of HIV among MSM in Seattle. Panel (**A**): Projected utilization of PrEP (blue dashed line) among all MSM in the absence of vaccine, compared to a potential decline in utilization of PrEP (blue solid line) corresponding to the entry of HIV vaccines (solid red line). The majority of PrEP use is among high‐risk men while HIV vaccines are used by low‐ and high‐risk men. Panel (**B**): Model estimates of the proportion of MSM living with HIV from 2004 to 2025, based on calibrated fit to available Seattle‐King County surveillance data. The black line projects the HIV prevalence up to 2045 in absence of vaccination as a reference for existing HIV treatment and prevention with PrEP. The coloured lines assume HIV vaccine with different efficacy becoming available starting in 2025. MSM, men who have sex with men; PrEP, pre‐exposure prophylaxis.

Daily oral Truvada^®^ (Gilead Sciences, Inc., Foster City, CA, USA), a combination product of 200 mg emtricitabine (FTC) and 300 mg tenofovir disoproxil fumarate (TDF), for PrEP is introduced in the model beginning in 2015. After 2014, the model assumes that 25% of high‐risk MSM and 6% of low‐risk MSM start using PrEP annually with 20% discontinuation rate. This assumption closely reproduces the expansion of PrEP usage in Seattle up to 2017 32 and is expected to result 25% overall PrEP coverage by 2025, with close to 50% of the high‐risk MSM using PrEP (see Figure 2A). Figure 2A visualizes the utilization rates of each product at a population level when all of the model inputs and assumptions are combined into the dynamic model. Evidence from completed efficacy trials shows that PrEP efficacy depends strongly on adherence 40, 41, 42, 43, 44, 45, 46, 47, 48. Results from most recent clinical studies 41, 42, conducted after Truvada has already proven efficacy, suggested that PrEP reduces the HIV risk by more than 80% which motivated the efficacy assumption in our model. Conservatively, we assumed that PrEP does not reduce infectivity once infected. Condom replacement, also known as risk compensation or behavioural disinhibition, is a decrease in condom use that may occur among people using biomedical HIV prevention modalities 46. In Seattle, on average, 63% of MSM sex acts are protected by a condom; while using PrEP, only 12.5% of MSM sex acts are protected by condoms 32. We do not include a disutility to account for PrEP adverse events, assuming that individuals with intolerable side effects would discontinue its use.

#### 2.4.2 HIV vaccine

The model simulates vaccination with a five‐dose regimen of a canarypox‐based vaccine ALVAC‐HIV vCP2438 DNA prime (Sanofi Pasteur, Paris, France) and bivalent gp120 protein subunit boost with MF59^®^ adjuvant (GSK, Brentford, UK) beginning in 2025 (Table S1) 13. The durability of vaccine protection is expected to wane over time 49, 50. We assume an average efficacy of 50% reduction in risk of infection lasting five years in duration 51, 52. We simulate vaccination campaigns every five years with coverage of 90% for those on PrEP and 60% for those who are not on PrEP (Figure 2A). We incorporate condom replacement among vaccinated MSM who continue to use PrEP but not to vaccinated non‐users. We assume the vaccine has no disutility and that future technologies in HIV testing will overcome any previously reported social risks from vaccine‐induced sero‐positivity 53, 54.

#### 2.4.3 Interactions between PrEP and vaccine

The model explores potential interactions among HIV vaccines, PrEP and condom use that risk mitigation of clinical and economic impact. We assume the protection from dual‐use of PrEP and vaccine is multiplicative. Condom use may decrease as PrEP use increases. The demand for PrEP may decrease when another biomedical HIV prevention choice is on the market and HIV vaccine utilization increases. We explore the following utilizations of PrEP and HIV vaccine delivery: (i) vaccine licensure in 2025; (ii) PrEP is targeted to high‐risk MSM while the vaccine is targeted to all MSM; (iii) HIV vaccination campaign cover 60% of low‐risk MSM every five years beginning in 2025 (red line in Figure 2A); (iv) PrEP users being three times more likely to receive an HIV vaccine; (v) after vaccination PrEP users continue on PrEP for an additional year. This hypothetical interaction was specified by soliciting a collection of expert opinions about plausible changes expected in utilization rate.

### 2.5 Approach to health outcomes

The following metrics of effectiveness are evaluated for each scenario over 20 years of intervention: cumulative number and fraction of new HIV infections prevented, reduction in HIV prevalence, and quality‐adjusted life years (QALYs) gained due to the vaccine programme. Person‐time in each health state is multiplied by the corresponding preference‐based utility weight, discounted 3% annually, and summed over the time horizon to calculate total QALYs 55, 56, 57, 58, 59.

### 2.6 Approach to costing

Costing of HIV prevention services follows a unit costing approach, also known as ingredients‐based, while the cost of HIV treatment relies on published studies based on aggregate healthcare costs. The cost of a clinic visit for HIV prevention services with risk reduction counselling is based on Centers for Medicare and Medicaid Services reimbursement rates for the corresponding relative value units in the Physician Fee Schedule January 2018 release 60. Medication costs reflect the Veterans Affairs National Acquisition Center Federal Supply Schedule (FSS) prices from March 2018.

PrEP costs include medication, quarterly clinic visits, testing for HIV and STIs, and other routine laboratory tests for monitoring (Table 1). The launch price of an HIV vaccine is unknown. Experts suggest benchmarking on the price of a recombinant human papillomavirus (HPV) vaccine because they similarly prevent transmission of a sexually transmitted virus. The FSS price for GARDASIL‐9 (Merck & Co., Inc., Kenilworth, NJ, USA), was $210 per dose in June 2019 61. While the HPV vaccine is delivered to adults in a two‐ or three‐dose series, ongoing Phase IIB clinical trials of HIV vaccines are testing a five‐dose series of vaccinations. To benchmark an estimate of the launch price for an HIV vaccine series, consultation with expert opinion assumed a 30% higher cost than the FSS price for a three‐dose series of the HPV vaccine, totaling $820 per series of HIV vaccine as the input for the main analysis. The cost of a clinic visit for HIV prevention counselling and laboratory tests for STIs is added to the vaccine price at each dose.

### 2.7 Sensitivity analysis

Parameter uncertainty was evaluated in a sensitivity analysis 34, 62, 63, 64. One‐way sensitivity analyses evaluated the effect of uncertainty from individual parameters. Scenario analysis evaluated multi‐way parameter uncertainty. To understand how robust the estimates of outcomes were to the choice of calibration parameters, we performed an uncertainty analysis using 100 calibration sets of inputs within a plausible range varying with respect to one another. We conducted a two‐way threshold analysis of HIV vaccine and PrEP prices to understand the maximum cost‐effective price for each product in relation to the price of the other.

## 3 Results

Maintaining the current trends of PrEP use, rates of diagnoses, linkage to care, treatment, and viral suppression, our analysis estimates 3074 new HIV infections between 2025 and 2045. We project an HIV prevalence of 7.7% among MSM living in Seattle in 2045, a decrease from 13.9% in 2018. On the path to this decline in prevalence, the model projects almost 15,000 HIV‐uninfected men using PrEP in 2045, double the number in 2018, resulting in the partial protection in one‐third of MSM.

HIV vaccines are projected to decrease the number of new infections, lower HIV prevalence and gain QALYs (Table 2). Seattle's HIV prevalence in 2045 would be 1.4 percentage points lower with 37.9% of new HIV infections avoided. Considering the imperfect protection of both PrEP and vaccine, the model projects 1164 new HIV infections would be avoided. Our simulations suggest that more than 600,000 vaccine doses will be needed to secure that at least 50% of the susceptible MSM population is protected over 20 years. This produces 63% fewer MSM using PrEP in 2045 compared to the same year with no vaccine.

**Table 2:** Model results

Outcome	Current practice	HIV vaccine, 50% efficacy	Incremental difference	Relative difference (%)^a^
HIV burden				
New HIV infections, 2025‐2045	3074	1910	−1164	−37.9%
New HIV diagnoses 2025‐2045	2934	2121	−814	−27.7%
People living with HIV in 2045	4806	3949	−857	−17.8%
HIV prevalence (%) in 2045	7.7%	6.3%	−1.4%	−18.0%
Utilization of biomedical prevention				
Protected by PrEP in 2025	11,233	11,233	0	0.0%
Total protected by PrEP or vaccine in 2045	14,905	36,680	21,775	146.1%
PrEP alone (% of susceptible)	14,905	5494	−9412	−63.1%
HIV vaccine alone (% of susceptible)	0	31,158	31,158	
PrEP + HIV vaccine (% of susceptible)	0	29	29	
Health outcomes				
Total LYs^b^	1,100,665	1,102,750	2086	0.2%
Total QALYs	923,770	924,486	717	0.1%
Costs				
Total cost (millions $)	$2396	$2426	$30	1.3%
PrEP costs (millions $)	$675	$224	−$450	−66.7%
HIV vaccine costs (millions $)	$0	$532	$532	
HIV care costs (millions $)	$1720	$1669	−$51	−3.0%
ICER ($ per QALY)			$42,473	

Costs are presented in a common currency of 2017 USD. Cost‐effectiveness analysis uses time horizon of 2025‐2045. Per capita and per capita susceptible calculations are based on the common population size of MSM projected in 2025.

ICER, incremental cost‐effectiveness ratio; LYs, life years; MSM, men who have sex with men; PrEP, pre‐exposure prophylaxis; QALYs, quality‐adjusted life years.

a The relative difference in HIV prevalence is slightly different from the relative difference in number of people living with HIV 2045 as more MSM are alive in 2045 with the vaccine – contributing to the denominator of HIV prevalence but not the number of cases living with HIV

b LY and QALYs summed among MSM ages 15‐64 years between the years 2025‐2045.

With model parameters based on US cost data over a 20‐year time horizon, the total incremental cost of introducing an HIV vaccine in Seattle is estimated to be $30 million from a healthcare sector perspective. Given the assumed rate of product substitution, PrEP costs would decrease by $450 million and the cost of $532 million on HIV vaccines would be introduced (Table 2). In addition, prevention with a 50% effective vaccine is expected to reduce HIV treatment costs by $51 million.

### 3.1 Cost‐effectiveness

**Figure 3:**
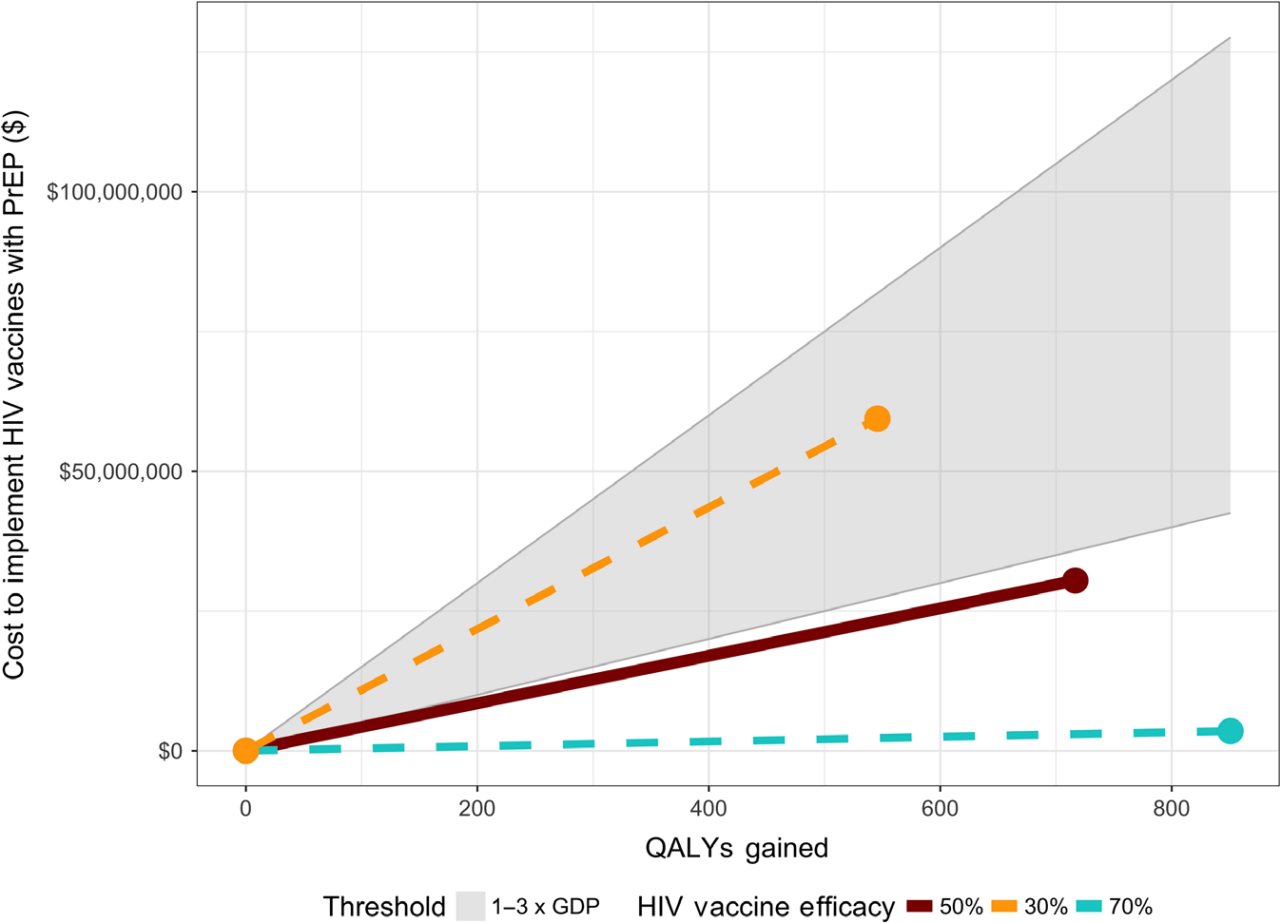
Cost‐effectiveness results. Scenario lines show additional cost of HIV vaccines against QALYs gained for ranges of vaccine efficacy. Shaded grey area represents the cost‐effectiveness threshold of 1‐3 times GDP per capita ($50,000 per QALY (lower edge)‐$150,000 per QALY (upper edge)). GDP, gross domestic product; PrEP, pre‐exposure prophylaxis; QALYs, quality‐adjusted life years.

The introduction of an HIV vaccine was predicted to have an incremental cost‐effectiveness ratio (ICER) of $42,473 per QALY gained. The cost per HIV infection avoided was $26,151. Scenarios in Figure 3 report the additional cost of HIV vaccines against QALYs gained, over time, highlighting each five‐year increment since start of the intervention. The shaded grey area reflects the cost‐effectiveness threshold range of 1‐3 times gross domestic product (GDP) per capita.

### 3.2 Sensitivity analysis

The greatest source of uncertainty and driver of cost‐effectiveness is HIV vaccine efficacy. Vaccine efficacy of 70% had an ICER of $4136 per QALY while 30% was $108,824 per QALY compared to no vaccine (Table 3). Vaccine cost‐effectiveness was also sensitive to the price of PrEP drugs. If competition from the entry of generic PrEP products reduces the medication cost to half by 2025, then the addition of an HIV vaccine would cost $336,671 per QALY gained compared to no vaccine. Assuming no condom displacement with HIV vaccines or PrEP lowered the ICER to $21,457 per QALY. The magnitude of health benefit from a vaccine was sensitive to the degree of condom replacement assumed when partially protected by PrEP and/or vaccine.

**Table 3:** Sensitivity analysis

Scenario	Inc Cost	Inc QALYs	ICER ($/QALY)
Main analysis	$30,439,907	717	$42,473
HIV vaccine 70% efficacy	$3,518,673	851	$4136
HIV vaccine 30% efficacy	$59,386,518	546	$108,824
No condom replacement	$12,135,182	566	$21,457
PrEP half price	$241,288,044	717	$336,671
HIV vaccine half price	−$180,422,645	717	Dominant
PrEP price doubled	−$391,256,368	717	Dominant
Doubled price HIV vaccine	$452,165,011	717	$630,908

Incremental results represent Seattle population‐level health gains and healthcare payer costs during the period 2025‐2045. Costs in 2017 USD. Dominant scenarios gained health and had lower cost than the reference comparator of PrEP with no HIV vaccine.

ICER, incremental cost‐effectiveness ratio; PrEP, pre‐exposure prophylaxis; QALYs, quality‐adjusted life years.

## 4 Discussion

This study used a dynamic transmission model to evaluate the cost‐effectiveness of an HIV vaccine launch in 2025, assuming the vaccine would complement and substitute some PrEP use. The tradeoffs from competition between two imperfect biomedical HIV prevention products include (i) the opportunity to vaccinate a larger fraction of the population than with PrEP alone and (ii) the downside from potential substitution with a less effective prevention product. Assuming most high‐risk and some low‐risk MSM in Seattle are using PrEP in 2025, rapid uptake of HIV vaccine by 60% of men, and declines in PrEP use with increasing vaccine uptake, we found an ICER of $42,473 per QALY gained compared to PrEP with no vaccine. In this case, HIV vaccines would be cost‐effective using a 1× GDP or 3× GDP per capita, cost‐effectiveness threshold. Though HIV vaccines increased total healthcare costs by $240 million, some costs were offset by reduction in HIV treatment and PrEP medications.

Key uncertainties in the analysis affect the results under different scenarios. As expected, scenarios with greater vaccine efficacy were more likely to find vaccines cost‐effective and led to a higher maximum threshold price where the vaccine would remain cost‐effective. Given that an HIV vaccine candidate is in development, the exact frequency of vaccine administrations will be determined with more data about the durability of protection. We have previously explored the impact of HIV vaccine durability and we have been examined implementation approaches for three‐year HIV vaccine campaigns in another study 65, 66. Results were also sensitive to assumptions about the rate of switching from PrEP to vaccines. If PrEP users who become vaccinated continued to use PrEP for the same length of time as non‐vaccinated PrEP users, the vaccine has a higher ICER and is therefore less likely to be cost‐effective. If all high‐risk men using PrEP switched immediately to a vaccine, the vaccine has a lower ICER and would be more likely to be cost‐effective, but the total population‐level health benefit is slightly smaller. Assuming no change in condom use among people using PrEP or vaccines produced a lower, more likely cost‐effective, ICER. Lastly, scenarios assuming generic PrEP prices in the future led to the vaccine being less likely cost‐effective, while alternative scenarios simulating the launch of newer, branded, long‐acting, injectable products for PrEP at higher prices affected the results by lowering the ICER for HIV vaccine introduction, meaning vaccines would be more likely to be cost‐effective.

This modelling analysis yields two important lessons: (i) the cost‐effectiveness of HIV vaccines will depend on the utilization and cost of PrEP at the time of launch and (ii) condom displacement with vaccines could diminish the potential benefit of vaccines for the population and lower its value. Even if, however, vaccines induce some condom displacement and decline in PrEP use, we project overall population health benefits. Policies guiding the interactions between these interventions could have substantial impact on the value of each product alone and in combination.

We compared the results of this model with two other published studies evaluating the cost‐effectiveness of HIV vaccines in the United States, and our conclusions were consistent when assuming the same vaccine price. A dynamic transmission model of HIV vaccines conducted in the pre‐PrEP era estimated an ICER of $91,000/QALY for universal HIV vaccination and net cost‐savings from targeting MSM when assuming a cost of $500 per vaccine series 24. A more recent static HIV model comparing PrEP offered with HIV vaccines to PrEP alone, assuming vaccines cost $2500 per series, also estimated a net cost‐savings for MSM 30. In our sensitivity analysis varying HIV vaccine costs, we similarly found vaccines costing $500 or $2500 per series would have a net cost‐savings for MSM.

There are several limitations to this modelling study that deserve mention. Alternative modelling structures, such as sexual network‐ or agent‐based models, could be developed to strengthen the assumption of structural sensitivity. Bernard and colleagues suggest, however, that among a set of specific model structures and parameter sets examined – when applied to this HIV vaccine question – produced similar cost‐effectiveness results, despite the differences in their structure 67. As all available epidemiological data were used for calibration, the model has not yet been validated for time periods outside 2004‐2014. The U.S. does not have a stated willingness to pay for health gains and we assumed a range of cost‐effectiveness thresholds from current recommendations 36, 37. Also, the generalizability of these findings should be limited to MSM in the US. Not only is the future cost of PrEP uncertain, the products that will be used for PrEP in the future are also uncertain. With no additional approvals of for new drugs or indications, we would expect generic TDF/FTC products to become available at lower cost. If newer PrEP products offer fewer side effects, this would effectively extend the patent period and prices are unlikely to decline. Tested model interactions between vaccine and PrEP utilization are limited and rely on plausible scenarios and assumptions described by expert opinion. Insurance status and PrEP medication cost were not found to be significant barriers for obtaining PrEP in one study 9, but price elasticity is not yet understood. Adverse events from PrEP and HIV vaccines are not well defined and estimating any related disutility is difficult.

The findings from this study point to several policy considerations. First, further public investment in US HIV vaccine clinical trials is warranted to reduce the uncertainty in expected vaccine efficacy. When regulatory bodies deem an HIV vaccine as having “good enough” efficacy, commercialization may be a challenge. This economic model does not include novel incentives that may be needed to encourage industry partners to commercialize and manufacture the product for global distribution. A target product profile to guide the innovation of better, cheaper, faster and point‐of‐care STI diagnostics may also be needed. Second, more research on and education to prevent a decrease in condom displacement with biomedical HIV prevention products is needed to optimize the potential effectiveness and prevent further outbreak of other STIs such as syphilis and gonorrhoea. Third, value‐based pricing of the vaccine at launch should consider both the risk level of the indicated population and the current cost of PrEP. Risk to public investment in immunization campaigns could be mitigated with outcomes‐based risk‐sharing agreements between government payers and the manufacturer with support from existing CDC surveillance systems.

## 5 Conclusions

Moderately effective HIV vaccines have the potential to be a cost‐effective intervention implemented alongside PrEP in Seattle. The potential population health gains from and value‐based price of an HIV vaccine in this setting depends on the degree of interaction and substitution with PrEP. Dual methods could be implemented as complementary products if lower PrEP pricing could be negotiated. Access to an HIV vaccine is desirable as it could increase the overall effectiveness of combination HIV prevention efforts and improve population health. HIV vaccines may have the potential to reach subpopulations that PrEP has been unable to reach. The barriers to implementation of and access to vaccines could be lower with provision at clinic visits compared to prescription drugs that require high adherence to be effective. Planning for the rollout and scale‐up of HIV vaccines should carefully consider the design of policies that guide interactions between potentially competing biomedical HIV prevention strategies.

## Competing interests

BA is a current employee at Flatiron Health, an independent subsidiary of Roche. JK is the Executive Director of the HVTN. DD is a Senior Staff Scientist working with both the HIV Prevention Trials Network and HVTN.

## Authors’ contributions

BA, DD, JC, RB, JK and LG contributed to study design. BA and DD modelled the study; BA, DD, JC, RB, JK and LG involved in analysis; BA, DD, JC, RB, JK and LG involved in interpretation of results. BA, DD, JC, RB, JK and LG involved in manuscript writing.

## Supporting information


**Appendix S1.** Supplementary material.
