# Supplementary material for: Competing biomedical HIV prevention strategies: potential cost‐effectiveness of HIV vaccines and PrEP in Seattle, WA
Source: J Int AIDS Soc. 2019 Aug 11;22(8):e25373. doi: 10.1002/jia2.25373 (PMC6689690; doi:10.1002/jia2.25373)
Supplement: Supplementary file 1 — Appendix S1. Supplementary material. [file JIA2-22-e25373-s001.docx]

**SUPPLEMENTARY APPENDIX**

Adamson et al, 2019


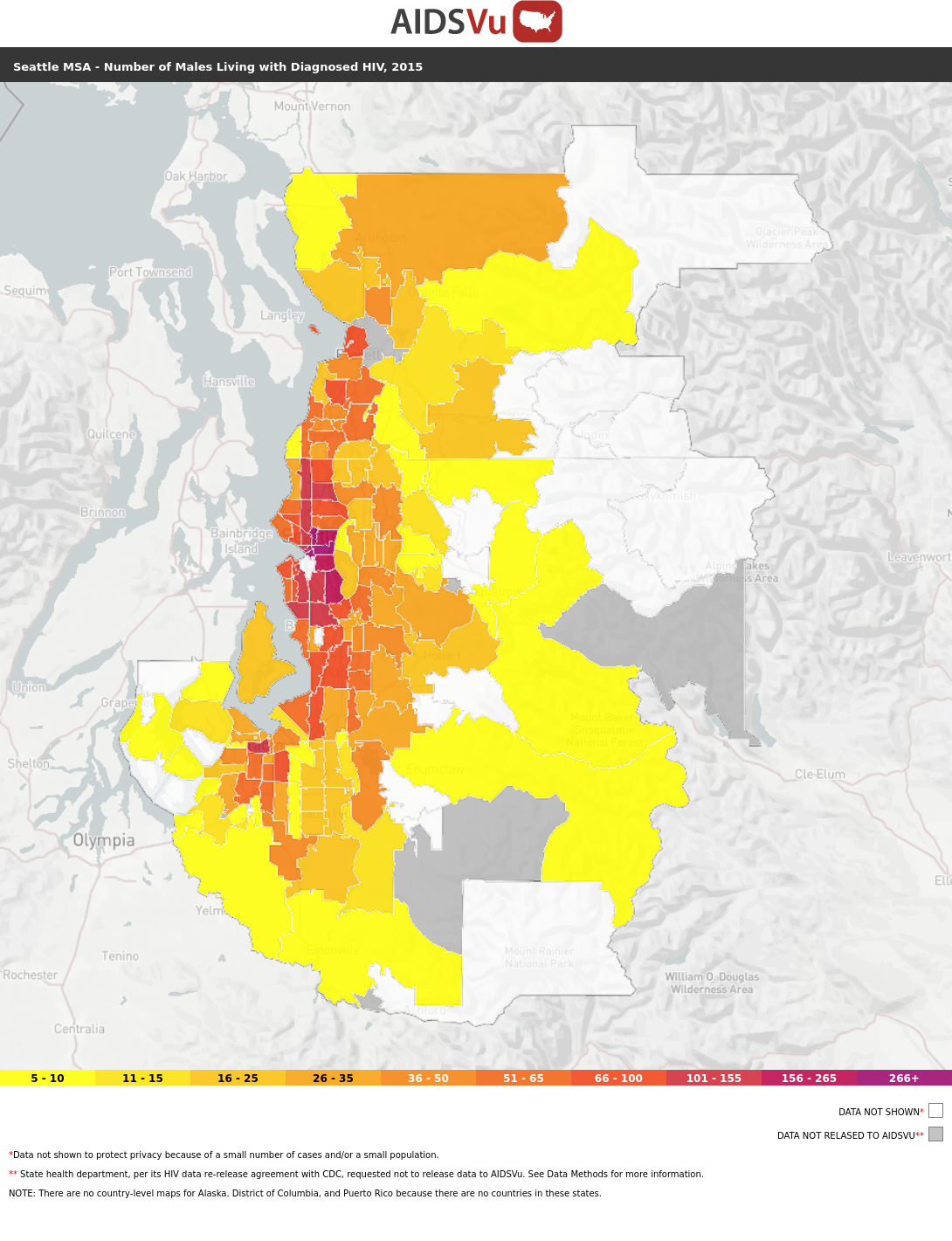


**Figure S1.** Number of males living with diagnosed HIV in Seattle, WA, 2015. *Source*: AIDSVu ^1^

**Table S1.** HIV vaccine candidate components, owners of intellectual property, and funders for the RV144 study in Thailand and HVTN 702 study in South Africa.

|  | **RV144 Thai Trial** | | |
| --- | --- | --- | --- |
| **Vaccine Component** | **DNA Prime** | **Protein Boost** | **Adjuvant** |
| **Description** | ALVAC-HIV recombinant canarypox vaccine, subtype B and E | AIDSVAX® B/E bivalent HIV gp120 envelope glycoprotein vaccine, subtypes B and E | 600 μg of alum adjuvant |
| **Owner** | Developed by Virogenetics Corporation (Troy, NY) and manufactured by Sanofi Pasteur (Marcy-l'Étoille, France) | Originally manufactured by Genentech, Inc., and further developed by VaxGen, Inc, (later acquired by Global Solutions for Infectious Diseases (San Francisco, CA)) | VaxGen, Inc, no IP |
| **Funder** | Supported in part by an Interagency Agreement (Y1-AI-2642-12) between the U.S. Army Medical Research and Materiel Command and the National Institute of Allergy and Infectious Diseases and by a cooperative agreement (W81XWH-07-2-0067) between the Henry M. Jackson Foundation for the Advancement of Military Medicine and the U.S. Department of Defense. Sanofi Pasteur provided the ALVAC-HIV vaccine, and Global Solutions for Infectious Diseases (VaxGen) provided the reagents for the immunogenicity assays. | | |

|  | **HVTN 702 South Africa Trial** | | |
| --- | --- | --- | --- |
| **Vaccine Component** | **DNA Prime** | **Protein Boost** | **Adjuvant** |
| **Description** | Canarypox-based vaccine ALVAC-HIV subtype C | bivalent gp120 protein subunit vaccine, subtype C | MF59 |
| **Owner** | Sanofi Pasteur | GSK | GSK |
| **Funder** | P5 members are NIAID, the Bill & Melinda Gates Foundation (BMGF), the South African Medical Research Council (SAMRC), HVTN, Sanofi Pasteur, GSK and the U.S. Military HIV Research Program. NIAID, BMGF and SAMRC fund the P5. The National Institute of Allergy and Infectious Diseases (NIAID) is sponsoring and funding HVTN 702. Sanofi Pasteur and GSK are providing the investigational vaccines for the trial. | | |

**Detailed Model Description**

Individuals are divided into risk groups ($i$: low risk <5 partners or high risk 5+ partners in past 12 months), age groups ($k$: young 15-24 years, middle 25-44 years, or older 45-64 years), and sexual role ($j$: insertive, receptive, or versatile position). The susceptible population $S_{q}$ is grouped by current use HIV prevention, where $q\in${1 (no protection), 2 (PrEP), 3 (vaccine), 4 (PrEP and vaccine)} represents coverage from a single or combination of products offering partial protection from infection.

The infected individuals are stratified by disease progression and where they fit into the HIV care cascade. Those who are infected but are unaware of their HIV status are in group $I_{\phi}$, where $\phi\in${1 (acute), 2 (CD4>500), 3 (CD4 350-500), 4 (CD4 200-349), 5 (CD4<200)} represents the disease stage of the individual stratified by CD4 count. Infected individuals are also assigned in compartments by treatment status as undiagnosed, diagnosed but not yet on ART, using ART but not virally unsuppressed, and virally suppressed while using ART. Those who are infected, diagnosed, but not engaged in care are in group $D_{\phi}$. Those who are engaged in care, but not on ART are in group $E_{\phi}$. Those who are on ART, but not virally suppressed due to lack of adherence or drug resistance are in group $U_{\phi}$. Finally, those who are virally suppressed from ART are in group $T_{\phi}$. We assume that PrEP and vaccination do not affect the course of infection. The rate of mixing between populations depends on the current distribution of risk, role, and age groups. We ensure the overall number of partnerships between groups remains balanced by continually updating the fraction of partners someone with a particular risk, role and age group has with the rest of the population.

We assume that it takes longer than three months to go through the care cascade and become virally suppressed. This means that newly suppressed people move from $U_{1}$ into $T_{2}$. We assume that those who are suppressed stay in their respective CD4 categories (so that if they stop treatment they return to their original unsuppressed state) but have the same behavior, infectivity, and utility across the CD4 dimension ($T_{2}-T_{5}$). Individuals on ART may interrupt treatment and reinitiate it again. Susceptible MSM who become sexually active join the community at constant rate, corresponding to estimated population growth among the MSM population in Seattle.^84^ The rates at which individuals acquire HIV infection depends on the annual number of partners per susceptible person, the number of sex acts per partnership, the fraction of sex acts protected by condoms, and the HIV acquisition risk per receptive and insertive anal intercourse with HIV infected partner.

### Model Equations

#### Parameters

In general:

- Risk status {1 (low risk), 2 (high risk)}
- Role status$j\in$ {1 (insertive), 2 (receptive), 3 (versatile)}
- Age status $k\in$ {1 (young), 2 (middle), 3 (old)}
- Prevention status $q\in$ { O (no biomedical prevention coverage), P (PrEP), V (vaccine), PV (PrEP and vaccine)}
- Disease stage {1 (acute), 2 (CD4>500), 3 (CD4 350-500), 4 (CD4 200-350), 5 (CD4<200)}
- Awareness status$A\in$ { I (Infected), P (Infected on PrEP), D (Diagnosed), E (Engaged in care), U (On ART, Unsuppressed), T (On ART, Suppressed)}

$\nu^{i}$: Fraction of new PrEP from susceptibles OFF PrEP with risk status $i$

$v_{q}$: Fraction of uninfected population vaccinated during 5-year campaigns starting 2025 by PrEP status $q$

$\omega_{q}$: Fraction of discontinuing PrEP from susceptibles ON PrEP with risk status $i$ and prevention status

$d_{k}$: Death rate (non-HIV related) for age

$\mu_{\phi}$: HIV-related death rate for disease stage

$a_{k}$: Aging rate from age $k$ to age

$\rho_{i,k}$: Fraction of population with risk status $i$ and in age group

$r_{j}$: Fraction of population with role status

$b$: Population birth (aging into population) rate

: Forward awareness transfer rate based on disease stage and awareness status $A$ (vertical flows)

: Drop rate from awareness status $A$ (losing suppression, dropping ART or leaving care) into disease stage

$\epsilon_{\Phi}^{A}$: Disease progression rate based on disease stage and awareness/treatment status $A$(horizontal flows)

$\lambda^{i,j,k}$: Force of infection for newly infected population entering

$\lambda_{P}^{i,j,k}$: Force of infection for newly infected population using PrEP entering

$\lambda_{V}^{i,j,k}$: Force of infection for newly infected vaccinated population entering $I_{1}^{i,j,k}$

$\lambda_{PV}^{i,j,k}$: Force of infection for newly infected vaccinated population using PrEP entering

$S_{q}^{i,j,k}$: Susceptible population with prevention status *q*, risk status $i$, role group $j$ and age group

: Infected population OFF PrEP with risk status $i$, role group $j$ and age group $k$ who are in disease stage

$I_{P,\phi}^{i,j,k}$: Infected population ON PrEP with risk status $i$, role group $j$ and age group $k$ who are in disease stage

: Diagnosed infected population with risk status $i$, role group $j$ and age group $k$ who are in disease stage

: Infected population who are engaged in care but not on ART with risk status $i$, role group $j$ and age group $k$ who are in disease stage

: Infected population who on ART but unsuppressed with risk status $i$, role group $j$ and age group $k$ who are in disease stage

: Infected population who on ART and suppressed with risk status $i$, role group $j$ and age group $k$ who are in disease stage

$I$: Infected population size $I=\sum_{i,j,k,\phi} \left( I_{\phi}^{i,j,k}+I_{P,\phi}^{i,j,k}+D_{\phi}^{i,j,k}+E_{\phi}^{i,j,k}+U_{\phi}^{i,j,k}+T_{\phi}^{i,j,k} \right)$. Here

$S$: Susceptible population size $S=\sum_{i,j,k,q} \left( S_{q}^{i,j,k} \right)$

$N$: Total population size$N=S+I$

δ_i,j_ : Kronecker delta function

#### Equations

For simplicity, all variables corresponding to age group have a value 0.

Between vaccination campaigns:

$\frac{dS^{i,j,k}}{dt}=b\delta_{1,i}r_{j}\rho_{i,k}N+\omega_{P}S_{P}^{i,j,k}+a_{k-1}\frac{\rho_{i,k}}{\sum_{l} \rho_{l,k}}\sum_{l} S^{l,j,k-1}-\left( d_{k}+\nu^{i}+a_{k} \right)S^{i,j,k}-\lambda^{i,j,k}S^{i,j,k},$

$\frac{dS_{P}^{i,j,k}}{dt}=\nu^{i}S^{i,j,k}+a_{k-1}\frac{\rho_{i,k}}{\sum_{l} \rho_{l,k}}\sum_{l} S_{P}^{l,j,k-1}-\left( d_{k}+\omega_{P}+a_{k} \right)S_{P}^{i,j,k}-\lambda_{P}^{i,j,k}S_{P}^{i,j,k},$

$\frac{dS_{V}^{i,j,k}}{dt}=\omega_{PV}S_{PV}^{i,j,k}+a_{k-1}\frac{\rho_{i,k}}{\sum_{l} \rho_{l,k}}\sum_{l} S_{V}^{l,j,k-1}-\left( d_{k}+a_{k} \right)S_{V}^{i,j,k}-\lambda_{V}^{i,j,k}S_{V}^{i,j,k},$

$\frac{dS_{PV}^{i,j,k}}{dt}=a_{k-1}\frac{\rho_{i,k}}{\sum_{l} \rho_{l,k}}\sum_{l} S_{PV}^{l,j,k-1}-\left( d_{k}+\omega_{PV}+a_{k} \right)S_{PV}^{i,j,k}-\lambda_{PV}^{i,j,k}S_{PV}^{i,j,k},$

$\frac{dI_{1}^{i,j,k}}{dt}=\lambda^{i,j,k}S^{i,j,k}+\lambda_{V}^{i,j,k}S_{V}^{i,j,k}+a_{k-1}\frac{\rho_{i,k}}{\sum_{l} \rho_{l,k}}\sum_{l} I_{1}^{l,j,k-1}-(d_{k}+\epsilon_{I,1}+a_{k})I_{1}^{i,j,k},$

$\frac{dI_{\phi}^{i,j,k}}{dt}=\epsilon_{I,\phi-1}I_{\phi-1}^{i,j,k}+a_{k-1}\frac{\rho_{i,k}}{\sum_{l} \rho_{l,k}}\sum_{l} I_{\phi}^{l,j,k-1}-(\mu_{\phi}+d_{k}+\epsilon_{I,\phi}+\sigma_{I,\phi}+a_{k})I_{\phi}^{i,j,k},$

$$\phi=2,\ldots,5$$

$\frac{dI_{P,1}^{i,j,k}}{dt}=\lambda_{P}^{i,j,k}S_{P}^{i,j,k}+\lambda_{PV}^{i,j,k}S_{PV}^{i,j,k}+a_{k-1}\frac{\rho_{i,k}}{\sum_{l} \rho_{l,k}}\sum_{l} I_{P,1}^{l,j,k-1}-(d_{k}+\epsilon_{P,1}+\sigma_{P,1}+a_{k})I_{P,1}^{i,j,k},$

$\frac{dI_{P,\phi}^{i,j,k}}{dt}=\epsilon_{P,\phi-1}I_{P,\phi-1}^{i,j,k}+a_{k-1}\frac{\rho_{i,k}}{\sum_{l} \rho_{l,k}}\sum_{l} I_{P,\phi}^{l,j,k-1}-(\mu_{\phi}+d_{k}+\epsilon_{P,\phi}+\sigma_{P,\phi}+a_{k})I_{P,\phi}^{i,j,k},$

$$\phi=2,\ldots,5$$

$\frac{dD_{1}^{i,j,k}}{dt}=\sigma_{P,1}I_{P,1}^{i,j,k}+a_{k-1}\frac{\rho_{i,k}}{\sum_{l} \rho_{l,k}}\sum_{l} D_{1}^{l,j,k-1}-(d_{k}+\epsilon_{D,1}+\sigma_{D,1}+a_{k})D_{1}^{i,j,k},$

$\frac{dD_{\phi}^{i,j,k}}{dt}=\epsilon_{D,\phi-1}D_{\phi-1}^{i,j,k}+\sigma_{P,\phi}I_{P,\phi}^{i,j,k}+\sigma_{I,\phi}I_{\phi}^{i,j,k}+\tau_{E,\phi}E_{\phi}^{i,j,k}+a_{k-1}\frac{\rho_{i,k}}{\sum_{l} \rho_{l,k}}\sum_{l} D_{\phi}^{l,j,k-1}$

$$-(\mu_{\phi}+d_{k}+\epsilon_{D,\phi}+\sigma_{D,\phi}+a_{k})D_{\phi}^{i,j,k},$$

$$\phi=2,\ldots,5$$

$\frac{dE_{1}^{i,j,k}}{dt}=\sigma_{D,1}D_{1}^{i,j,k}+a_{k-1}\frac{\rho_{i,k}}{\sum_{l} \rho_{l,k}}\sum_{l} E_{1}^{l,j,k-1}-(d_{k}+\epsilon_{E,1}+\sigma_{E,1}+a_{k})E_{1}^{i,j,k},$

$\frac{dE_{\phi}^{i,j,k}}{dt}=\epsilon_{E,\phi-1}E_{\phi-1}^{i,j,k}+\sigma_{D,\phi}D_{\phi}^{i,j,k}+\tau_{U,\phi}U^{i,j,k}+a_{k-1}\frac{\rho_{i,k}}{\sum_{l} \rho_{l,k}}\sum_{l} E_{\phi}^{l,j,k-1}$

$$-(\mu_{\phi}+d_{k}+\tau_{E,\phi}+\epsilon_{E,\phi}+\sigma_{E,\phi}+a_{k})E_{\phi}^{i,j,k},$$

$$\phi=2,\ldots,5$$

$\frac{dU_{1}^{i,j,k}}{dt}=\sigma_{E,1}E_{1}^{i,j,k}+a_{k-1}\frac{\rho_{i,k}}{\sum_{l} \rho_{l,k}}\sum_{l} U_{1}^{l,j,k-1}-(d_{k}+\epsilon_{U,1}+\sigma_{U,1}+a_{k})U_{1}^{i,j,k},$

$\frac{dU_{\phi}^{i,j,k}}{dt}=\epsilon_{U,\phi-1}U_{\phi-1}^{i,j,k}+\sigma_{E,\phi}E_{\phi}^{i,j,k}+\tau_{T,\phi}T_{\phi}^{i,j,k}+a_{k-1}\frac{\rho_{i,k}}{\sum_{l} \rho_{l,k}}\sum_{l} U_{\phi}^{l,j,k-1}$

$$-(\mu_{\phi}+d_{k}+\tau_{U,\phi}+\epsilon_{U,\phi}+\sigma_{U,\phi}+a_{k})U_{\phi}^{i,j,k},$$

$$\phi=2,\ldots,5$$

$\frac{dT_{2}^{i,j,k}}{dt}=\sigma_{U,1}U_{1}^{i,j,k}+\sigma_{U,2}U_{2}^{i,j,k}+a_{k-1}\frac{\rho_{i,k}}{\sum_{l} \rho_{l,k}}\sum_{l} T_{2}^{l,j,k-1}-(d_{k}+\tau_{T,\phi}+a_{k})T_{2}^{i,j,k},$

$\frac{dT_{\phi}^{i,j,k}}{dt}=\sigma_{U,\phi}U_{\phi}^{i,j,k}+a_{k-1}\frac{\rho_{i,k}}{\sum_{l} \rho_{l,k}}\sum_{l} T_{\phi}^{l,j,k-1}-(d_{k}+\tau_{T,\phi}+a_{k})T_{\phi}^{i,j,k},\quad\phi=3,4,5$

At the time of vaccination campaigns:

$${S_{0}^{i,j,k}=(1-v}_{0})(S_{0}^{i,j,k}+S_{V}^{i,j,k})$$

$${S_{V}^{i,j,k}=v}_{0}(S_{0}^{i,j,k}+S_{V}^{i,j,k})$$

$${S_{P}^{i,j,k}=(1-v}_{p})(S_{P}^{i,j,k}+S_{PV}^{i,j,k})$$

$${S_{PV}^{i,j,k}=v}_{p}(S_{P}^{i,j,k}+S_{PV}^{i,j,k})$$

#### Force of Infection

The number of new infections among the susceptible class *S^i,j,k^* (risk status *i*, role status *j* and age group *k*) due to contacts with the infected class $W_{\phi}^{x,y,z}$ (risk status *x*, role status *y* and age group *z* who are in disease stage ) are calculated using the following rule and accounting for the biomedical HIV protection method used (none, PrEP, vaccine or both) :

| Number susceptible in class *S^i,j,k^* | X | Estimated # partners for age group *k* with risk status *i* | X | likelihood that the partner is from age group *z* with risk status *x* and role status *y* | X | probability that the partner is infected in the awareness/ treatment state W and disease stage | X | annual transmission probability in partnership between  *S^i,j,k^* and $W_{\phi}^{x,y,z}$ |
| --- | --- | --- | --- | --- | --- | --- | --- | --- |

Partnership likelihood is based on mixing matrices by age, risk and role, i.e, proportion of partnership that a person from each group has with every other group. These matrices are updated at each step to balance the participating parties (see below). Age/risk mixing is assumed independent from role mixing.

As a result, the force of infection on a susceptible individual who is not vaccinated nor using PrEP is:

$\lambda^{i,j,k}=\eta^{i,k}\sum_{x,y,z} m_{r}^{i,k,x,z}m_{\rho}^{j,y}(\sum_{\phi} (1-(1-(1-\alpha_{c})\theta_{\phi}\beta)^{(1-\psi^{j,y})c_{0}{n_{\phi}n}^{i,x}}\cdot(1-\theta_{\phi}\beta)^{(1-\psi^{j,y})(1-c_{0}{{)n}_{\phi}n}^{i,x}}\cdot$

$$(1-(1-\alpha_{c})\theta_{R}\theta_{\phi}\beta)^{\psi^{j,y}c_{0}{n_{\phi}n}^{i,x}}\cdot(1-\theta_{R}\theta_{\phi}\beta)^{\psi^{j,y}(1-c_{0}{{)n}_{\phi}n}^{i,x}})\frac{\left( I_{\phi}^{x,y,z}+I_{P,\phi}^{x,y,z}+D_{\phi}^{x,y,z}+E_{\phi}^{x,y,z} \right)}{N^{x,y,z}}+$$

$$\sum_{\phi} (1-(1-(1-\alpha_{c})\theta_{\phi}\beta(1-\alpha_{U,art}))^{(1-\psi^{j,y})c_{0}{n_{\phi}n}^{i,x}}\cdot(1-\theta_{\phi}\beta(1-\alpha_{U,art}))^{(1-\psi^{j,y})(1-c_{0}{{)n}_{\phi}n}^{i,x}}\cdot$$

$$(1-(1-\alpha_{c})\theta_{R}\theta_{\phi}\beta(1-\alpha_{U,art}))^{\psi^{j,y}c_{0}{n_{\phi}n}^{i,x}}\cdot(1-\theta_{R}\theta_{\phi}\beta(1-\alpha_{U,art}))^{\psi^{j,y}(1-c_{0}{{)n}_{\phi}n}^{i,x}})\frac{U_{\phi}^{x,y,z}}{N^{x,y,z}}).$$

The force of infection on a PrEP user who is not vaccinated is:

$\lambda_{P}^{i,j,k}=\eta^{i,k}\sum_{x,y,z} m_{r}^{i,k,x,z}m_{\rho}^{j,y}(\sum_{\phi} (1-(1-(1-\alpha_{c})\theta_{\phi}\beta(1-\alpha_{p}))^{(1-\psi^{j,y})c_{P}{n_{\phi}n}^{i,x}}\cdot$

$$(1-\theta_{\phi}\beta(1-\alpha_{p}))^{(1-\psi^{j,y})(1-c_{P}{{)n}_{\phi}n}^{i,x}}\cdot(1-(1-\alpha_{c})\theta_{R}\theta_{\phi}\beta(1-\alpha_{p}))^{\psi^{j,y}c_{P}{n_{\phi}n}^{i,x}}\cdot$$

$$(1-\theta_{R}\theta_{\phi}\beta(1-\alpha_{p}))^{\psi^{j,y}(1-c_{P}{{)n}_{\phi}n}^{i,x}})\frac{\left( I_{\phi}^{x,y,z}+I_{P,\phi}^{x,y,z}+D_{\phi}^{x,y,z}+E_{\phi}^{x,y,z} \right)}{N^{x,y,z}}+$$

$$\sum_{\phi} (1-(1-(1-\alpha_{c})\theta_{\phi}\beta(1-\alpha_{p})(1-\alpha_{U,art}))^{(1-\psi^{j,y})c_{P}{n_{\phi}n}^{i,x}}\cdot$$

$$(1-\theta_{\phi}\beta(1-\alpha_{p})(1-\alpha_{U,art}))^{(1-\psi^{j,y})(1-c_{P}{{)n}_{\phi}n}^{i,x}}\cdot$$

$$(1-(1-\alpha_{c})\theta_{R}\theta_{\phi}\beta(1-\alpha_{p})(1-\alpha_{U,art}))^{\psi^{j,y}c_{P}{n_{\phi}n}^{i,x}}\cdot$$

$$(1-\theta_{R}\theta_{\phi}\beta(1-\alpha_{p})(1-\alpha_{U,art}))^{\psi^{j,y}(1-c_{P}{{)n}_{\phi}n}^{i,x}})\frac{U_{\phi}^{x,y,z}}{N^{x,y,z}}).$$

The force of infection on a susceptible individual who is vaccinated but not using PrEP is:The force of infection on an individual who is vaccinated but not using PrEP is:

$$\lambda_{PV}^{i,j,k}=\eta^{i,k}\sum_{x,y,z} m_{r}^{i,k,x,z}m_{\rho}^{j,y}(\sum_{\phi} (1-(1-(1-\alpha_{c})\theta_{\phi}\beta(1-\alpha_{p})(1-\alpha_{v}))^{(1-\psi^{j,y})c_{PV}n_{\phi}n^{i,x}}\cdot$$

$$(1-\theta_{\phi}\beta(1-\alpha_{p})(1-\alpha_{v}))^{(1-\psi^{j,y})(1-c_{PV})n_{\phi}n^{i,x}}\cdot(1-(1-\alpha_{c})\theta_{R}\theta_{\phi}\beta(1-\alpha_{p})(1-\alpha_{v}))^{\psi^{j,y}c_{PV}n_{\phi}n^{i,x}}\cdot$$

$$(1-\theta_{R}\theta_{\phi}\beta(1-\alpha_{p})(1-\alpha_{v}))^{\psi^{j,y}(1-c_{PV})n_{\phi}n^{i,x}})\frac{\left( I_{\phi}^{x,y,z}+I_{P,\phi}^{x,y,z}+D_{\phi}^{x,y,z}+E_{\phi}^{x,y,z} \right)}{N^{x,y,z}}+$$

$$\sum_{\phi} (1-(1-\alpha_{c})\theta_{\phi}\beta(1-\alpha_{p})(1-\alpha_{v})(1-\alpha_{U,art}))^{(1-\psi^{j,y})c_{PV}n_{\phi}n^{i,x}}\cdot$$

$$(1-\theta_{\phi}\beta(1-\alpha_{p})(1-\alpha_{v})(1-\alpha_{U,art}))^{(1-\psi^{j,y})(1-c_{PV})n_{\phi}n^{i,x}}\cdot$$

$$(1-(1-\alpha_{c})\theta_{R}\theta_{\phi}\beta(1-\alpha_{p})(1-\alpha_{v})(1-\alpha_{U,art}))^{\psi^{j,y}c_{PV}n_{\phi}n^{i,x}}\cdot$$

$$(1-\theta_{R}\theta_{\phi}\beta(1-\alpha_{p})(1-\alpha_{v})(1-\alpha_{U,art}))^{\psi^{j,y}(1-c_{PV})n_{\phi}n^{i,x}})\frac{U_{\phi}^{x,y,z}}{N^{x,y,z}}).$$

Here:

$\eta^{i,k}$: number of partners for individuals in risk group $i$ and age group ,

*n^i,x^* : number of acts per year in partnership between risk group $i$ and risk group $x$,

$n_{\phi}$: relative sexual activity (multiplier for the number of acts per year) in partnership in which the infected partner is in disease stage to account for reduced sexual activity during late HIV stages using CD4 >500 as a reference,

$m_{\rho}^{i,k,x,z}$: probability for a partner of a person with risk status $i$ from age group $k$ to be with risk status $x$from age group $z$(risk/age mixing),

$m_{r}^{j,y}$: mixing probability between people with role $j$ and role $y$ (role mixing),

$\alpha_{c}$: condom efficacy in reducing susceptibility per act,

$c_{q}$: rate of condom use by uninfected individuals with prevention status *q*,

: relative HIV transmission risk per act by disease stage , using asymptomatic stage (CD4 >500) as a reference

$\beta$: HIV-transmission risk per unprotected insertive anal act from untreated infected MSM in asymptomatic stage (CD4>500) to uninfected MSM not using PrEP or vaccine,

$\psi^{j,y}$: fraction of receptive acts when MSM with role status *j* is partnering with MSM with role status *y*,

$\theta_{R}$: relative HIV acquisition risk per receptive anal act compared to insertive anal acts,

$\alpha_{U,art}$: ART efficacy in reducing infectiousness per act when virally unsuppressed,

$\alpha_{p}$: PrEP efficacy in reducing susceptibility per act,

$\alpha_{v}$: Vaccine efficacy in reducing susceptibility per act,

$N^{x,y,z}$: Population size with risk status *x*, role status *y*. and age group *z* . Here $T_{1}^{x,y,z}=0.$

### Partnership Balancing

### *Balancing by age and risk*

We consider mixing between all possible 6 age/risk groups. Likelihood of partnerships between 2 age/risk groups are stored in 6x6 age/risk mixing matrix ***A=a_ij_*** representing the likelihood that each partner of an individual from group *i* to be from group *j*. ***A*** is informed initially by data representative for the MSM population in Seattle. The matrix is constantly updated to balance the number of partnerships between different age/risk groups. The procedure aims to equilize the number of people participating at each end of the partnerships between 2 different age/risk groups (*i* and *j*) calculated as:

(Number of people in group *i*)*(Number of partners per individual)*(Likelihood *a_ij_*)

At each time step, the balancing procedure proceeds as follows:

1. All off-diagonal pairs *a_ij_* and *a_ij_* are adjusted to equilize the number of people participating at each end of the partnerships. Adjusted is the entry which leads to decrease in the sum of the off-diagonal values
2. Diagonal entries are adjusted to guarantee that each row sums up to 1.

Step 1) of the procedure guarantees that the sum of the off-diagonal entries decreases and therefore remains below 1. This makes step 2) always possible. The procedure favors sexual mixing within each age/risk group which is supported by self-reported behavioral data.

**Table S2.** Initial value of the mixing matrix used in the simulations

| Mixing likelihoods | Young, low risk | Middle, low risk | Old,  low risk | Young, high risk | Middle, high risk | Old,  high risk |
| --- | --- | --- | --- | --- | --- | --- |
| Young, low risk | 0.2394 | 0.0986 | 0.1925 | 0.2066 | 0.0751 | 0.1878 |
| Middle, low risk | 0.1323 | 0.254 | 0.2222 | 0.0899 | 0.127 | 0.1746 |
| Old,  low risk | 0.1666 | 0 | 0.381 | 0.1111 | 0 | 0.3413 |
| Young, high risk | 0.1231 | 0.0313 | 0.0901 | 0.3217 | 0.1176 | 0.3162 |
| Middle, high risk | 0.0899 | 0.1013 | 0.0727 | 0.1396 | 0.3327 | 0.2638 |
| Old,  high risk | 0.0806 | 0 | 0.1573 | 0.2258 | 0 | 0.5363 |

### *Balancing by role*

Fractions of partners by role are constantly updated to balance the number of partnerships between different role groups.

We use the following likelihood matrix of partnering between sexual role groups:

**Table S3.** Sexual partnership mixing matrix

| Role group | Receptive | Insertive | Versatile |
| --- | --- | --- | --- |
| Receptive | a | b | 1-a-b |
| Insertive | x | c | 1-c-x |
| Versatile | y | z | 1-y-z |

Assuming that the overall number of partners per year is the same for each role group our balancing procedure requires that:

x*N_ins_= b*N_rec_

y*N_ver_= (1-a-b) *N_rec_

z*N_ver_= (1-c-x) *N_ins_

In all simulations, the values of a, b and c remain constant while x, y and z are updated at each time step. Simulations which result in negative mixing rates are discarded.

**Model Inputs**

**Table S4. Complete list of fixed parameter values used in the analysis**

| Variable name | Description | Value |
| --- | --- | --- |
| N_0_ | Initial population size in 2004 | 45000 |
|  | Initial fraction of infected population who are on ART | 0.83 |
|  | Initial fraction of the infected population on ART who are virally suppressed | 0.83 |
| ν^1^ | Fraction of low risk population starting PrEP annually after 2014 | 0.06 |
| ν^2^ | Fraction of high risk population starting PrEP annually after 2014 | 0.25 |
| $v_{0}$ | Fraction of uninfected population vaccinated during 5-year campaigns starting 2025 | 0.6 |
| $v_{P}$ | Fraction of PrEP users vaccinated during 5-year campaigns starting 2025 | 0.9 |
| ω_P_ | Annual rate of dropping PrEP if unvaccinated | 0.2 |
| ω_PV_ | Annual rate of dropping PrEP if vaccinated | 1 |
| *1/a_1_* | Duration of the young age group (15-24) | 10 years |
| *1/a_2_* | Duration of the middle age age group (25-44) | 20 years |
| *1/a_3_* | Duration of the old age group (45-64) | 20 years |
| used in ρ_i,1_ | Fraction of the population in the young age group | 0.168 |
| used in ρ_i,2_ | Fraction of the population in the middle age group | 0.463 |
| used in ρ_i,1_ | Fraction of MSM in the young age group with high risk status | 0.31 |
| used in ρ_i,2_ | Fraction of MSM in the middle age group with high risk status | 0.099 |
| used in ρ_i,3_ | Fraction of MSM in the old age group with high risk status | 0.065 |
| *d_1_* | Death rate (non-HIV related) for the young age group | 0.001319 |
| *d_2_* | Death rate (non-HIV related) for the middle age group | 0.001574 |
| *d_3_* | Death rate (non-HIV related) for the oldage group | 0.008438 |
| used in *r_1_* | Fraction of MSM with insertive role status | 0.3248 |
| used in *r_2_* | Fraction of MSM with versatile role group | 0.2683 |
| *b* | Population recruitment rate (MSM turning 15) | 0.03 |
| *σ_P,ϕ_* | Annual rate of HIV screening for PrEP users | 2 |
| *σ_I,1_* | Annual rate of HIV diagnosis for acutely infected MSM, unvaccinated, not using PrEP | 0 |
| *σ_I,2-4_* | Rate of HIV diagnosis for infected MSM with CD4 > 200 | Calculated to match overall rate |
| 1/ϵ_A,1_, A in {I, P} | Duration of acute stage | 0.33 years |
| 1/ϵ_A,2_, A in {I,P,D,E,U} | Duration of HIV stage with CD4>500 | 3.32 years |
| 1/ϵ_A,3_, A in {I,P,D,E,U} | Duration of HIV stage with CD4 350-500 | 2.7 years |
| 1/ϵ_A,4_, A in {I,P,D,E,U} | Duration of HIV stage with CD4 200-350 | 5.5 years |
| 1/ϵ_A,5_, A in {I,P,D,E,U} | Duration of HIV stage with CD4<200 | 5.06 years |
| μ_2_ | HIV-related death rate for MSM with CD4>500, off ART | 0.0032 |
| μ_3_ | HIV-related death rate for MSM with CD4 350-500, off ART | 0.0039 |
| μ_4_ | HIV-related death rate for MSM with CD4 200-350, off ART | 0.009 |
| η^2,1^ | Annual number of partners for MSM in the young age group with high risk status | 10.5 |
| η^2,2^ | Annual number of partners for MSM in the middle age group with high risk status | 10.5 |
| η^2,3^ | Annual number of partners for MSM in the old age group with high risk status | 6 |
| η^1,1^ | Annual number of partners for MSM in the young age group with low risk status | 1.5 |
| η^1,2^ | Annual number of partners for MSM in the middle age group with low risk status | 1.5 |
| η^1,3^ | Annual number of partners for MSM in the old age group with low risk status | 1 |
| θ_5_ | Multiplier for reduced sexual activity when partner is in AIDS stage (CD4<200) | 0.75 |
| *m*^1,1^ | Probability for someone in role group "insertive" to partner with someone in role group "insertive" | 0.18 |
| *m*^1,3^ | Probability for someone in role group "receptive" to partner with someone in role group "insertive" | 0.6 |
| *m*^3,3^ | Probability for someone in role group "receptive" to partner with someone in role group "receptive" | 0.08 |
| *c_0_* and *c_V_* | Fraction of acts which are protected by a condom when PrEP is not used | 0.63 |
| *c_P_* and *c_PV_* | Fraction of acts of PrEP users, protected by a condom | 0.125 |
| θ_1_ | Relative infectiousness of acutely infected MSM compared to MSM with CD4>200 | 26 |
| θ_5_ | Relative infectiousness of MSM with CD4<200 compared to MSM with CD4>200 | 3 |
| ψ^1,1^ | Fraction of receptive acts in partnership between two MSM with insertive role status | 0.5 |
| ψ^1,2^ | Fraction of receptive acts when MSM with insertive role status is partnering with MSM with receptive role status | 0 |
| ψ^1,3^ | Fraction of receptive acts when MSM with insertive role status is partnering with MSM with versatile role status | 0 |
| ψ^2,1^ | Fraction of receptive acts when MSM with receptive role status is partnering with MSM with insertive role status | 1 |
| ψ^2,2^ | Fraction of receptive acts in partnership between two MSM with receptive role status | 0.5 |
| ψ^2,3^ | Fraction of receptive acts when MSM with receptive role status is partnering with MSM with versatile role status | 1 |
| ψ^3,1^ | Fraction of receptive acts when MSM with versatile role status is partnering with MSM with insertive role status | 1 |
| ψ^3,2^ | Fraction of receptive acts for MSM with versatile role status partnering MSM with receptive role status | 0 |
| ψ^3,3^ | Fraction of receptive acts in partnership between two MSM with versatile role status | 0.5 |
|  | ART efficacy in reducing infectiousness per act when virally suppressed | 1 |
| α_p_ | PrEP efficacy in reducing susceptibility per act | 0.8 |
| α_v_ | Vaccine efficacy in reducing susceptibility per act | 0.5 |

**Model Calibration**

The model simulations start in 2004, parameterized with values listed in Table 1 and Table S4. We calibrated the model outcomes to match data from 2012 to select parameter sets that best captures the epidemic trends and clinical disease progression among MSM in King County, WA.^36,70^ We fit model outcomes to the HIV prevalence and the treatment cascade (% diagnosed, % engaged in care, % on ART, % virally suppressed). Monte Carlo filtering was used to select 100 parameter sets for which all targets are within calibrated ranges listed in Table 1 of the main text. The procedure of parameter sets selection consist of the following:

1. All uncertain parameters are sampled from their ranges in Table S5 and HIV prevalence target is checked against its 2012 target range
2. If HIV prevalence is accepted, then all awareness/treatment parameters (#1 - #12 and #21-#25 in Table S5) are resampled while keeping behavioral and transmission parameters fixed (#13 - #20 in Table S5). This step is repeated up to 50 times while all HIV prevalence and treatment cascade outcomes are within targeted ranges.
3. The procedure is repeated while 100 parameter sets are selected

**Table S5. List of parameters ranges used in model calibration**

| # | Variable name | Description | Range |
| --- | --- | --- | --- |
| 1 | *σ_I,5_* | Annual rate of HIV diagnosis for infected MSM with CD4<200 in absence of intervention | 0.2-0.4 |
| 2 |  | Overall rate of HIV diagnosis for infected MSM annually | 0.2-0.3 |
| 3 | *σ_D,2-4_* | Annual rate of engagement in care for acutely diagnosed MSM with CD4>200 | 0.5-1 |
| 4 | *σ_D,5_* | Annual rate of engagement in care for acutely diagnosed MSM with CD4<200 | 0.5-1 |
| 5 | *σ_E,2-5_* | Annual rate of ART initiation for MSM in care | 0.5-1 |
| 6 | *σ_U,2-4_* | Annual rate of achieving viral suppression on ART for MSM with CD4>200 | 2.0-6.0 |
| 7 | *σ_U,5_* | Annual rate of achieving viral suppression on ART for MSM with CD4<200 | 1.0-3.0 |
| 8 | *τ_E,2-4_* | Rate at which engaged in care MSM with CD4>200 drop from care | 0.2-0.3 |
| 9 | *τ_E,5_* | Rate at which engaged in care MSM with CD4<200 drop from care | 0.1-0.2 |
| 10 | *τ_U,2-5_* | Annual ART drop rate | 0.2-0.25 |
| 11 | *τ_T,2-5_* | Rate of losing virally suppression due to inconsistent use of ART | 0.2-0.3 |
| 12 | used in ϵ_U,i_ | Multiplicative factor which extends duration of HIV stages while on ART with detectible viral load (virally unsuppressed) | 1-1.5 |
| 13 | used in η^2,k^ | Multiplicative factor which adjusts for overreported (overlapping) partnerships of high-risk MSM | 0.5-1 |
| 14 | used in η^1,k^ | Multiplicative factor which adjusts for overreported (overlapping) partnerships of low-risk MSM | 0.78-1 |
| 15 | *n*^1,1^ | Number of acts per year in partnership in which both MSM have low risk status | 40-60 |
| 16 | *n*^1,2^ | Number of acts per year in partnership between MSM with low risk status | 2.0-5.0 |
| 17 | *n*^2,2^ | Number of acts per year in partnership in which both MSM have high risk status | 1.0-2.0 |
| 18 | α_c_ | Condom efficacy in reducing infectiousness per act | 0.7-0.9 |
| 19 | β | HIV-transmission probability per unprotected insertive anal act with untreated infected MSM with CD4>200 | 0.001-0.002 |
| 20 | θ_R_ | Relative HIV acquisition risk per receptive sex act compared to insertive act | 3.0-7.0 |
| 21 | $\boldsymbol{\alpha}_{\boldsymbol{U,art}}$ | ART efficacy in reducing infectiousness per act when virally unsuppressed | 0.3-0.7 |
| 22 |  | HIV prevalence in 2004 | 0.14-0.16 |
| 23 |  | Initial fraction of infected MSM (CD4>200) who are diagnosed | 0.8-0.9 |
| 24 |  | Initial fraction of infected MSM (CD4<200) who are diagnosed | 0.94-0.98 |
| 25 |  | Initial fraction of diagnosed MSM who are engaged in care | 0.8-0.9 |

Given the starting population size of 45,000 MSM in 2004, the model estimates growth to 62,388 MSM by 2045.

**Table S6.** Description of health states and corresponding utility weight

| **Health**  **State** | **HIV status** | **Aware**  **Status** | **Stage of Care** | **cd4cat** | **CD4** | **Risk**  **Level** | **ART** | **Utility**  **Weight** |
| --- | --- | --- | --- | --- | --- | --- | --- | --- |
| SL | susceptible | S | Susceptible | NA | NA | NA | NA | 1.00 |
| SH | susceptible | S | Susceptible | NA | NA | NA | NA | 1.00 |
| SPL | susceptible | S | Susceptible | NA | NA | NA | NA | 1.00 |
| SPH | susceptible | S | Susceptible | NA | NA | NA | NA | 1.00 |
| SVL | susceptible | S | Susceptible | NA | NA | NA | NA | 1.00 |
| SVH | susceptible | S | Susceptible | NA | NA | NA | NA | 1.00 |
| SPVL | susceptible | S | Susceptible | NA | NA | NA | NA | 1.00 |
| SPVH | susceptible | S | Susceptible | NA | NA | NA | NA | 1.00 |
| I1L | infected | I | Infected and unaware | 1 | Acute | low | 0 | 0.69 |
| I1H | infected | I | Infected and unaware | 1 | Acute | high | 0 | 0.69 |
| I2L | infected | I | Infected and unaware | 2 | >500 | low | 0 | 0.73 |
| I2H | infected | I | Infected and unaware | 2 | >500 | high | 0 | 0.73 |
| I3L | infected | I | Infected and unaware | 3 | 350-500 | low | 0 | 0.71 |
| I3H | infected | I | Infected and unaware | 3 | 350-500 | high | 0 | 0.71 |
| I4L | infected | I | Infected and unaware | 4 | 200-349 | low | 0 | 0.69 |
| I4H | infected | I | Infected and unaware | 4 | 200-349 | high | 0 | 0.69 |
| I5L | infected | I | Infected and unaware | 5 | <200 | low | 0 | 0.69 |
| I5H | infected | I | Infected and unaware | 5 | <200 | high | 0 | 0.69 |
| D1L | infected | D | Diagnosed | 1 | Acute | low | 0 | 0.69 |
| D1H | infected | D | Diagnosed | 1 | Acute | high | 0 | 0.69 |
| D2L | infected | D | Diagnosed | 2 | >500 | low | 0 | 0.73 |
| D2H | infected | D | Diagnosed | 2 | >500 | high | 0 | 0.73 |
| D3L | infected | D | Diagnosed | 3 | 350-500 | low | 0 | 0.71 |
| D3H | infected | D | Diagnosed | 3 | 350-500 | high | 0 | 0.71 |
| D4L | infected | D | Diagnosed | 4 | 200-349 | low | 0 | 0.69 |
| D4H | infected | D | Diagnosed | 4 | 200-349 | high | 0 | 0.69 |
| D5L | infected | D | Diagnosed | 5 | <200 | low | 0 | 0.69 |
| D5H | infected | D | Diagnosed | 5 | <200 | high | 0 | 0.69 |
| E1L | infected | E | Enaged (not on ART) | 1 | Acute | low | 0 | 0.69 |
| E1H | infected | E | Enaged (not on ART) | 1 | Acute | high | 0 | 0.69 |
| E2L | infected | E | Enaged (not on ART) | 2 | >500 | low | 0 | 0.73 |
| E2H | infected | E | Enaged (not on ART) | 2 | >500 | high | 0 | 0.73 |
| E3L | infected | E | Enaged (not on ART) | 3 | 350-500 | low | 0 | 0.71 |
| E3H | infected | E | Enaged (not on ART) | 3 | 350-500 | high | 0 | 0.71 |
| E4L | infected | E | Enaged (not on ART) | 4 | 200-349 | low | 0 | 0.69 |
| E4H | infected | E | Enaged (not on ART) | 4 | 200-349 | high | 0 | 0.69 |
| E5L | infected | E | Enaged (not on ART) | 5 | <200 | low | 0 | 0.69 |
| E5H | infected | E | Enaged (not on ART) | 5 | <200 | high | 0 | 0.69 |
| U1L | infected | U | Unsuppressed and ON ART | 1 | Acute | low | 1 | 0.69 |
| U1H | infected | U | Unsuppressed and ON ART | 1 | Acute | high | 1 | 0.69 |
| U2L | infected | U | Unsuppressed and ON ART | 2 | >500 | low | 1 | 0.73 |
| U2H | infected | U | Unsuppressed and ON ART | 2 | >500 | high | 1 | 0.73 |
| U3L | infected | U | Unsuppressed and ON ART | 3 | 350-500 | low | 1 | 0.71 |
| U3H | infected | U | Unsuppressed and ON ART | 3 | 350-500 | high | 1 | 0.71 |
| U4L | infected | U | Unsuppressed and ON ART | 4 | 200-349 | low | 1 | 0.69 |
| U4H | infected | U | Unsuppressed and ON ART | 4 | 200-349 | high | 1 | 0.69 |
| U5L | infected | U | Unsuppressed and ON ART | 5 | <200 | low | 1 | 0.69 |
| U5H | infected | U | Unsuppressed and ON ART | 5 | <200 | high | 1 | 0.69 |
| T1L | infected | T | Suppressed | 1 | Acute | low | 1 | 0.73 |
| T1H | infected | T | Suppressed | 1 | Acute | high | 1 | 0.73 |
| T2L | infected | T | Suppressed | 2 | >500 | low | 1 | 0.73 |
| T2H | infected | T | Suppressed | 2 | >500 | high | 1 | 0.73 |
| T3L | infected | T | Suppressed | 3 | 350-500 | low | 1 | 0.73 |
| T3H | infected | T | Suppressed | 3 | 350-500 | high | 1 | 0.73 |
| T4L | infected | T | Suppressed | 4 | 200-349 | low | 1 | 0.73 |
| T4H | infected | T | Suppressed | 4 | 200-349 | high | 1 | 0.73 |
| T5L | infected | T | Suppressed | 5 | <200 | low | 1 | 0.73 |
| T5H | infected | T | Suppressed | 5 | <200 | high | 1 | 0.73 |

**Table S7.** Modeled population sizes at the start and end of the time horizon

| **Case** | **Start**  **2004** | **Year**  **2025** | **End**  **2045** | **Growth (no.)** | **Percent**  **Growth** |
| --- | --- | --- | --- | --- | --- |
| Reference: PrEP alone | 45,000 | 51606 | 62388 | 17389 | 0.3864 |
| Intervention: Vaccine + PrEP | 45,000 | 51606 | 62532 | 17532 | 0.3896 |

###


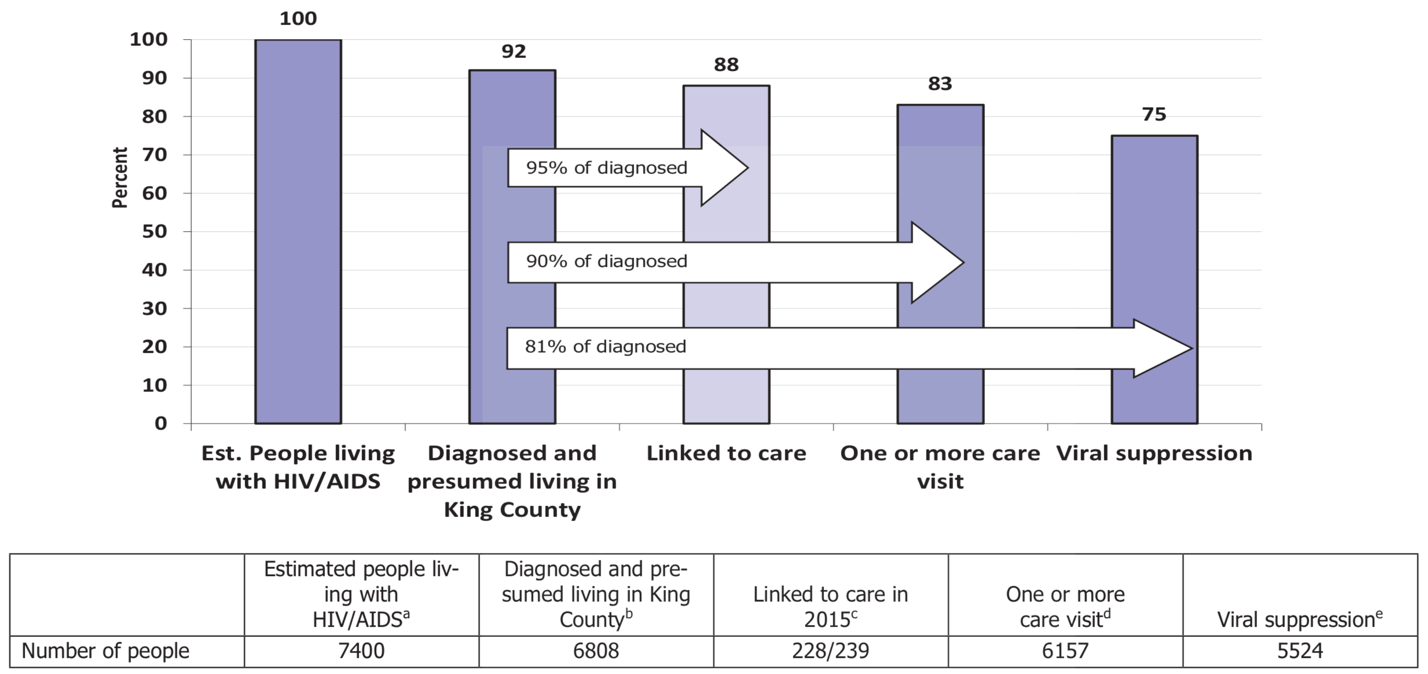


**Figure S2.** King County HIV Care Continuum in 2015

*Source*: Seattle Public Health King County, (data reported through of June 30, 2016)

^a^ Percent undiagnosed was calculated as 6% among MSM for King County; prior estimate of 15% was used for non-MSM (based on CDC and Washington State estimates) resulting in an estimate of 7.3% overall, rounded up to 8% for a slightly more conservative estimate (this may be the most uncertain bar in the continuum). Estimated people living with HIV/AIDS is calculated by dividing “diagnosed and presumed living in King County” residents by 0.92.

^b^ Diagnosed cases are those presumed living in King County during 2015. Individuals with no contact for ten or more years were presumed to have relocated or died (N=249). Others with unconfirmed deaths or relocations (identified, for example by online Internet database searches, but not confirmed by the new jurisdiction or another secondary source) and no laboratory results reported for >18 months were also excluded (N=161).

^c^ Linked to care in 2015 is not a subset of earlier data (hence different color in the graph) and is based on the percent diagnosed in 2015 with a CD4 or viral load test within 3 months of diagnosis. The percent linked in the figure, 88%, is the percent of diagnosed cases in 2015 who linked (95.4%) times 92.0% to account for undiagnosed cases.

^d^ One or more care visit was based on one or more reported laboratory result (CD4, viral load, genotype).

^e^ Viral suppression is defined as the most recent viral load test result in 2015 less than 200 copies.

### Estimating Cost-Effectiveness

We evaluate ICERs using a cost-effectiveness threshold ranging from 1-3x the GDP per capita, or approximately $50,000 - $150,000 per QALY gained using the equation

$ICER=\frac{Cost_{intervention}-Cost_{standardcare}}{QALYs_{intervention}-QALYs_{standardcare}}.$

### Approach to Health Outcomes

Health impact is measured in HIV infections averted and total QALYs gained, both discounted 3% annually.^62,88^ The estimation of compartment size mid-point moments each quarter represent units of one-quarter person-year time. This “life year” matrix integrates with a function to adjust for health-state specific utility weights and discount 3% annually. The sum of the resulting “QALY matrix” from 2025-2045 represents the total population-level discounted QALYs for a simulation. To calculate average QALYs per person in a model with fluctuating population size, we sum the number of people alive in each year of the model and divide by the time horizon length to find the average population size for the simulation. In summary, person-time in each health state multiplied by the corresponding preference-based utility weights will be summed and discounted 3% annually to estimate total QALYs.^53–57^

**Sensitivity Analysis**

**Table S8.** Scenario with 70% vaccine efficacy

| **Outcome** | **Reference** | **Vaccine** | **Incremental** |
| --- | --- | --- | --- |
| HIV BURDEN —————- |  |  |  |
| New Infections 2025-2045 | 3,074 | 1,380 | -1,694 |
| Diagnoses 2025-2045 | 2,935 | 1,672 | -1,263 |
| PLWH 2045 | 4,806 | 3,557 | -1,249 |
| HIV Prevalence (%) 2045 | 7.70% | 5.68% | -2.02% |
| UTILIZATION ————— |  |  |  |
| PrEP Users in 2025 | 11,233 | 11,233 | 0 |
| Total Protected in 2045 | 14,905 | 36,987 | 22,081 |
| —PrEP alone | 14,905 | 5,523 | -9,383 |
| —HIV vaccine alone | 0 | 31,435 | 31,435 |
| —PrEP and HIV vaccine | 0 | 29 | 29 |
| OUTCOMES —————— |  |  |  |
| Life Years, total | 1,100,665 | 1,103,765 | 3,100 |
| QALYs, total | 923,770 | 924,620 | 851 |
| COSTS ——————— |  |  |  |
| Total Cost, $ | $2,396,128,051 | $2,399,646,724 | $3,518,673 |
| —PrEP cost, $ | $675,392,497 | $225,458,175 | -$449,934,322 |
| —Vaccine cost, $ | $0 | $534,016,765 | $534,016,765 |
| —HIV Care Cost, $ | $1,720,735,553 | $1,640,171,783 | -$80,563,770 |
| ICER, $/QALY |  |  | $4,136 |

**Table S9.** Scenario with 30% vaccine efficacy

| **Outcome** | **Reference** | **Vaccine** | **Incremental** |
| --- | --- | --- | --- |
| HIV BURDEN —————- |  |  |  |
| New Infections 2025-2045 | 3,074 | 2,517 | -556 |
| Diagnoses 2025-2045 | 2,935 | 2,630 | -305 |
| PLWH 2045 | 4,806 | 4,408 | -398 |
| HIV Prevalence (%) 2045 | 7.70% | 7.06% | -0.65% |
| UTILIZATION ————— |  |  |  |
| PrEP Users in 2025 | 11,233 | 11,233 | 0 |
| Total Protected in 2045 | 14,905 | 36,322 | 21,417 |
| —PrEP alone | 14,905 | 5,461 | -9,445 |
| —HIV vaccine alone | 0 | 30,832 | 30,832 |
| —PrEP and HIV vaccine | 0 | 29 | 29 |
| OUTCOMES —————— |  |  |  |
| Life Years, total | 1,100,665 | 1,101,670 | 1,005 |
| QALYs, total | 923,770 | 924,315 | 546 |
| COSTS ——————— |  |  |  |
| Total Cost, $ | $2,396,128,051 | $2,455,514,569 | $59,386,518 |
| —PrEP cost, $ | $675,392,497 | $224,315,801 | -$451,076,697 |
| —Vaccine cost, $ | $0 | $530,444,101 | $530,444,101 |
| —HIV Care Cost, $ | $1,720,735,553 | $1,700,754,667 | -$19,980,886 |
| ICER, $/QALY |  |  | $108,824 |

**Table S10.** Scenario with no condom displacement

| **Outcome** | **Reference** | **Vaccine** | **Incremental** |
| --- | --- | --- | --- |
| HIV BURDEN —————- |  |  |  |
| New Infections 2025-2045 | 3,074 | 1,827 | -1,347 |
| Diagnoses 2025-2045 | 2,935 | 2,030 | -983 |
| PLWH 2045 | 4,806 | 3,842 | -964 |
| HIV Prevalence (%) 2045 | 7.70% | 6.14% | -1.56% |
| UTILIZATION ————— |  |  |  |
| PrEP Users in 2025 | 11,233 | 11,273 | 0 |
| Total Protected in 2045 | 14,905 | 36,770 | 21,865 |
| —PrEP alone | 14,905 | 5,506 | -9,399 |
| —HIV vaccine alone | 0 | 31,234 | 31,234 |
| —PrEP and HIV vaccine | 0 | 29 | 29 |
| OUTCOMES —————— |  |  |  |
| Life Years, total | 1,100,665 | 1,103,521 | 2,857 |
| QALYs, total | 923,770 | 924,335 | 566 |
| COSTS ——————— |  |  |  |
| Total Cost, $ | $2,396,128,051 | $2,408,263,232 | $12,135,182 |
| —PrEP cost, $ | $675,392,497 | $225,534,454 | -$449,858,043 |
| —Vaccine cost, $ | $0 | $533,545,002 | $533,545,002 |
| —HIV Care Cost, $ | $1,720,735,553 | $1,649,183,776 | -$71,551,777 |
| ICER, $/QALY |  |  | $21,457 |

**Table S11.** Scenario with half priced PrEP

| **Outcome** | **Reference** | **Vaccine** | **Incremental** |
| --- | --- | --- | --- |
| HIV BURDEN —————- |  |  |  |
| New Infections 2025-2045 | 3,074 | 1,910 | -1,164 |
| Diagnoses 2025-2045 | 2,935 | 2,121 | -814 |
| PLWH 2045 | 4,806 | 3,949 | -857 |
| HIV Prevalence (%) 2045 | 7.70% | 6.31% | -1.39% |
| UTILIZATION ————— |  |  |  |
| PrEP Users in 2025 | 11,233 | 11,233 | 0 |
| Total Protected in 2045 | 14,905 | 36,680 | 21,775 |
| —PrEP alone | 14,905 | 5,494 | -9,412 |
| —HIV vaccine alone | 0 | 31,158 | 31,158 |
| —PrEP and HIV vaccine | 0 | 29 | 29 |
| OUTCOMES —————— |  |  |  |
| Life Years, total | 1,100,665 | 1,102,750 | 2,086 |
| QALYs, total | 923,770 | 924,486 | 717 |
| COSTS ——————— |  |  |  |
| Total Cost, $ | $2,080,009,965 | $2,321,298,010 | $241,288,044 |
| —PrEP cost, $ | $359,274,412 | $119,641,363 | -$239,633,049 |
| —Vaccine cost, $ | $0 | $532,299,370 | $532,299,370 |
| —HIV Care Cost, $ | $1,720,735,553 | $1,669,357,277 | -$51,378,276 |
| ICER, $/QALY |  |  | $336,671 |

**Table S12**. Scenario with half priced HIV vaccine

| **Outcome** | **Reference** | **Vaccine** | **Incremental** |
| --- | --- | --- | --- |
| HIV BURDEN —————- |  |  |  |
| New Infections 2025-2045 | 3,074 | 1,910 | -1,164 |
| Diagnoses 2025-2045 | 2,935 | 2,121 | -814 |
| PLWH 2045 | 4,806 | 3,949 | -857 |
| HIV Prevalence (%) 2045 | 7.70% | 6.31% | -1.39% |
| UTILIZATION ————— |  |  |  |
| PrEP Users in 2025 | 11,233 | 11,233 | 0 |
| Total Protected in 2045 | 14,905 | 36,680 | 21,775 |
| —PrEP alone | 14,905 | 5,494 | -9,412 |
| —HIV vaccine alone | 0 | 31,158 | 31,158 |
| —PrEP and HIV vaccine | 0 | 29 | 29 |
| OUTCOMES —————— |  |  |  |
| Life Years, total | 1,100,665 | 1,102,750 | 2,086 |
| QALYs, total | 923,770 | 924,486 | 717 |
| COSTS ——————— |  |  |  |
| Total Cost, $ | $2,396,128,051 | $2,215,705,405 | -$180,422,645 |
| —PrEP cost, $ | $675,392,497 | $224,911,311 | -$450,481,187 |
| —Vaccine cost, $ | $0 | $321,436,818 | $321,436,818 |
| —HIV Care Cost, $ | $1,720,735,553 | $1,669,357,277 | -$51,378,276 |
| ICER, $/QALY |  |  | -$251,745 |

**Table S13.** Scenario with PrEP price doubled

| **Outcome** | **Reference** | **Vaccine** | **Incremental** |
| --- | --- | --- | --- |
| HIV BURDEN —————- |  |  |  |
| New Infections 2025-2045 | 3,074 | 1,910 | -1,164 |
| Diagnoses 2025-2045 | 2,935 | 2,121 | -814 |
| PLWH 2045 | 4,806 | 3,949 | -857 |
| HIV Prevalence (%) 2045 | 7.70% | 6.31% | -1.39% |
| UTILIZATION ————— |  |  |  |
| PrEP Users in 2025 | 11,233 | 11,233 | 0 |
| Total Protected in 2045 | 14,905 | 36,680 | 21,775 |
| —PrEP alone | 14,905 | 5,494 | -9,412 |
| —HIV vaccine alone | 0 | 31,158 | 31,158 |
| —PrEP and HIV vaccine | 0 | 29 | 29 |
| OUTCOMES —————— |  |  |  |
| Life Years, total | 1,100,665 | 1,102,750 | 2,086 |
| QALYs, total | 923,770 | 924,486 | 717 |
| COSTS ——————— |  |  |  |
| Total Cost, $ | $3,028,364,221 | $2,637,107,853 | -$391,256,368 |
| —PrEP cost, $ | $1,307,628,668 | $435,451,206 | -$872,177,462 |
| —Vaccine cost, $ | $0 | $532,299,370 | $532,299,370 |
| —HIV Care Cost, $ | $1,720,735,553 | $1,669,357,277 | -$51,378,276 |
| ICER, $/QALY |  |  | -$545,922 |

**Table S14.** Scenario with HIV vaccine price doubled

| **Outcome** | **Reference** | **Vaccine** | **Incremental** |
| --- | --- | --- | --- |
| HIV BURDEN —————- |  |  |  |
| New Infections 2025-2045 | 3,074 | 1,910 | -1,164 |
| Diagnoses 2025-2045 | 2,935 | 2,121 | -814 |
| PLWH 2045 | 4,806 | 3,949 | -857 |
| HIV Prevalence (%) 2045 | 7.70% | 6.31% | -1.39% |
| UTILIZATION ————— |  |  |  |
| PrEP Users in 2025 | 11,233 | 11,233 | 0 |
| Total Protected in 2045 | 14,905 | 36,680 | 21,775 |
| —PrEP alone | 14,905 | 5,494 | -9,412 |
| —HIV vaccine alone | 0 | 31,158 | 31,158 |
| —PrEP and HIV vaccine | 0 | 29 | 29 |
| OUTCOMES —————— |  |  |  |
| Life Years, total | 1,100,665 | 1,102,750 | 2,086 |
| QALYs, total | 923,770 | 924,486 | 717 |
| COSTS ——————— |  |  |  |
| Total Cost, $ | $2,396,128,051 | $2,848,293,062 | $452,165,011 |
| —PrEP cost, $ | $675,392,497 | $224,911,311 | -$450,481,187 |
| —Vaccine cost, $ | $0 | $954,024,474 | $954,024,474 |
| —HIV Care Cost, $ | $1,720,735,553 | $1,669,357,277 | -$51,378,276 |
| ICER, $/QALY |  |  | $630,908 |

**References**

1. Emory University Rollins School of Public Health. AIDSVu. www.aidsvu.org.

2. U.S. Census Bureau Population Division. *National Population Projection, 2014*. Washington, DC; 2015.

3. HIV/AIDS Epidemiology Unit, Public Health - Seattle & King County, and Infectious Diseases and Reproductive Health Assessment Unit, Washington State Department of Health. *HIV/AIDS Epidemiology Report*. 2005;65:1-33.

4. Sagaon-teyssier L, Suzan-monti M, Demoulin B, Capitant C, Lorente N. Uptake of PrEP and condom and sexual risk behavior among MSM during the ANRS IPERGAY trial. 2016;28:48-55. doi:10.1080/09540121.2016.1146653

5. Seattle WS, County K, Allen E, et al. EPIDEMIOLOGY REPORT. 2015.

6. Institute for Clinical and Economic Review. *ICER Value Assessment Framework Summary*. Boston; 2015.

7. Gold M, Siegel J, Russell L, Weinstien M. *Cost-Effectiveness in Health and Medicine*. New York, New York: Oxford University Press; 1996.

8. Briggs A, Claxton K, Sculpher M. *Decision Modelling for Health Economic Evaluation*. 1st ed. London: Oxford University Press; 2006.

9. Joyce VR, Barnett PG, Bayoumi AM, et al. Health-related quality of life in a randomized trial of antiretroviral therapy for advanced HIV disease. *Journal of acquired immune deficiency syndromes (1999)*. 2009;50(1):27-36. doi:10.1097/QAI.0b013e31818ce6f3

10. Kauf TL, Roskell N, Shearer A, et al. A predictive model of health state utilities for HIV patients in the modern era of highly active antiretroviral therapy. *Value in health : the journal of the International Society for Pharmacoeconomics and Outcomes Research*. 2008;11(7):1144-1153. doi:10.1111/j.1524-4733.2008.00326.x

11. Bayoumi AM, Barnett PG, Joyce VR, et al. Cost-effectiveness of newer antiretroviral drugs in treatment-experienced patients with multidrug-resistant HIV disease. *Journal of acquired immune deficiency syndromes (1999)*. 2013;64(4):382-391. doi:10.1097/QAI.0000000000000002

12. Sanders GD, Bayoumi AM, Sundaram V, et al. Cost-effectiveness of screening for HIV in the era of highly active antiretroviral therapy. *The New England journal of medicine*. 2005;352(6):570-585. doi:10.1056/NEJMsa042657

13. Tengs TO, Lin TH. A meta-analysis of utility estimates for HIV/AIDS. *Medical decision making : an international journal of the Society for Medical Decision Making*. 2002;22(6):475-481.
