# Supplementary material for: Competing biomedical HIV prevention strategies: potential cost‐effectiveness of HIV vaccines and PrEP in Seattle, WA
Source: J Int AIDS Soc. 2019 Aug 11;22(8):e25373. doi: 10.1002/jia2.25373 (PMC6689690; doi:10.1002/jia2.25373)
Supplement: Supplementary file 2 [file JIA2-22-e25373-s002.pdf]

SUPPLEMENTARY APPENDIX

Adamson et al, 2019

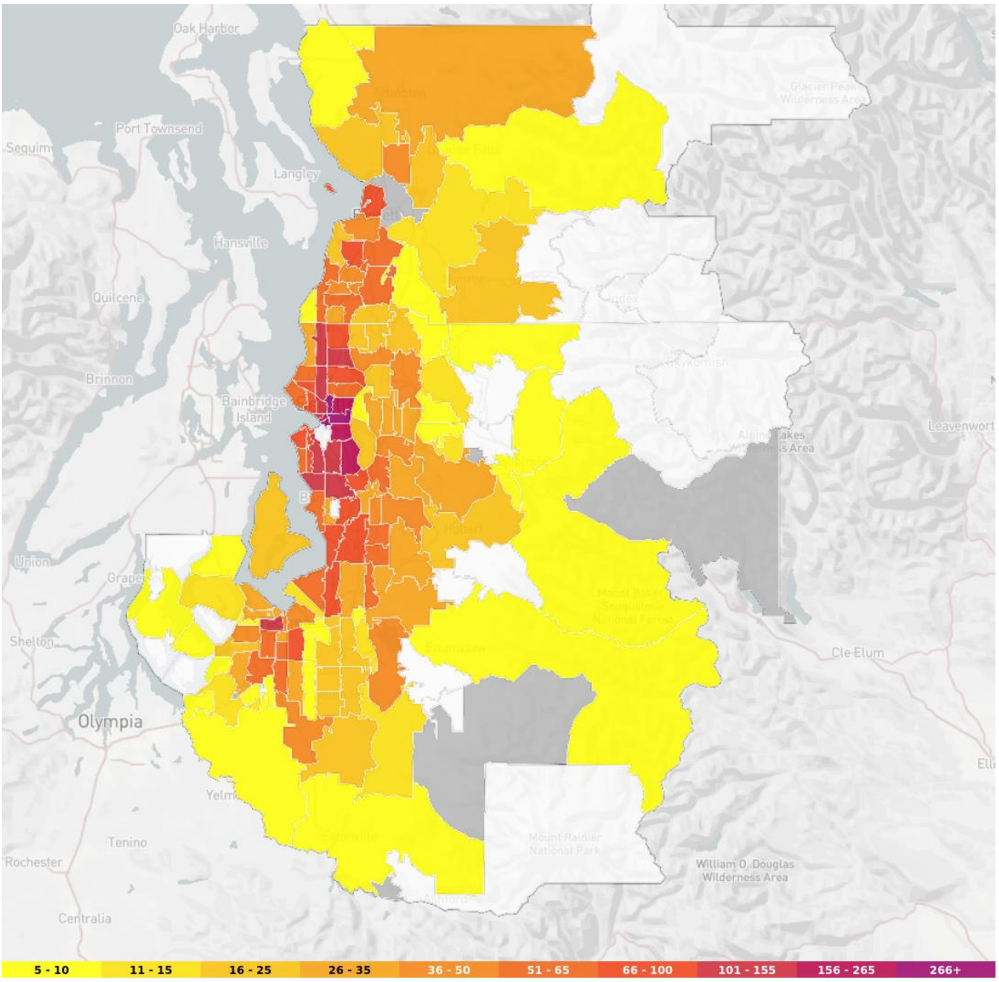

Figure S1. Number of males living with diagnosed HIV in Seattle, WA, 2015. *Source*: AIDSVu [1]

Table S1. HIV vaccine candidate components, owners of intellectual property, and funders for the RV144 study in Thailand and HVTN 702 study in South Africa.

	RV144 Thai Trial		
**Vaccine Component**	**DNA Prime**	**Protein Boost**	**Adjuvant**
**Description**	ALVAC-HIV recombinant canarypox vaccine, subtype B and E	AIDSVAX® B/E bivalent HIV gp120 envelope glycoprotein vaccine, subtypes B and E	600 µg of alum adjuvant
**Owner**	Developed by Virogenetics Corporation (Troy, NY) and manufactured by Sanofi Pasteur (Marcy-l'Étoille, France)	Originally manufactured by Genentech, Inc., and further developed by VaxGen, Inc, (later acquired by Global Solutions for Infectious Diseases (San Francisco, CA))	VaxGen, Inc, no IP
**Funder**	Supported in part by an Interagency Agreement (Y1-AI-2642-12) between the U.S. Army Medical Research and Materiel Command and the National Institute of Allergy and Infectious Diseases and by a cooperative agreement (W81XWH-07-2-0067) between the Henry M. Jackson Foundation for the Advancement of Military Medicine and the U.S. Department of Defense. Sanofi Pasteur provided the ALVAC-HIV vaccine, and Global Solutions for Infectious Diseases (VaxGen) provided the reagents for the immunogenicity assays.		

	HVTN 702 South Africa Trial		
**Vaccine Component**	**DNA Prime**	**Protein Boost**	**Adjuvant**
**Description**	Canarypox-based vaccine ALVAC-HIV subtype C	bivalent gp120 protein subunit vaccine, subtype C	MF59
**Owner**	Sanofi Pasteur	GSK	GSK
**Funder**	P5 members are NIAID, the Bill & Melinda Gates Foundation (BMGF), the South African Medical Research Council (SAMRC), HVTN, Sanofi Pasteur, GSK and the U.S. Military HIV Research Program. NIAID, BMGF and SAMRC fund the P5. The National Institute of Allergy and Infectious Diseases (NIAID) is sponsoring and funding HVTN 702. Sanofi Pasteur and GSK are providing the investigational vaccines for the trial.		

Detailed Model Description

Individuals are divided into risk groups (i: low risk <5 partners or high risk 5+ partners in past 12 months), age groups (k : young 15-24 years, middle 25-44 years, or older 45-64 years), and sexual role (j : insertive, receptive, or versatile position). The susceptible population S_q is grouped by current use HIV prevention, where $q \in \{1$ (no protection), 2 (PrEP), 3 (vaccine), 4 (PrEP and vaccine)$\}$ represents coverage from a single or combination of products offering partial protection from infection.

Field Code Changed

Field Code Changed

Field Code Changed

The infected individuals are stratified by disease progression and where they fit into the HIV care cascade. Those who are infected but are unaware of their HIV status are in group I_ϕ, where $\phi \in \{1$ (acute), 2 (CD4>500), 3 (CD4 350-500), 4 (CD4 200-349), 5 (CD4<200)$\}$ represents the disease stage of the individual stratified by CD4 count. Infected individuals are also assigned in compartments by treatment status as undiagnosed, diagnosed but not yet on ART, using ART but not virally unsuppressed, and virally suppressed while using ART. Those who are infected, diagnosed, but not engaged in care are in group D_ϕ. Those who are engaged in care, but not on ART are in group E_ϕ. Those who are on ART, but not virally suppressed due to lack of adherence or drug resistance are in group U_ϕ. Finally, those who are virally suppressed from ART are in group T_ϕ. We assume that PrEP and vaccination do not affect the course of infection. The rate of mixing between populations depends on the current distribution of risk,

role, and age groups. We ensure the overall number of partnerships between groups remains balanced by continually updating the fraction of partners someone with a particular risk, role and age group has with the rest of the population.

We assume that it takes longer than three months to go through the care cascade and become virally suppressed. This means that newly suppressed people move from U_1 into T_2. We assume that those who are suppressed stay in their respective CD4 categories (so that if they stop treatment they return to their original unsuppressed state) but have the same behavior, infectivity, and utility across the CD4 dimension ($T_2 - T_5$). Individuals on ART may interrupt treatment and reinitiate it again. Susceptible MSM who become sexually active join the community at constant rate, corresponding to estimated population growth among the MSM population in Seattle.[84] The rates at which individuals acquire HIV infection depends on the annual number of partners per susceptible person, the number of sex acts per partnership, the fraction of sex acts protected by condoms, and the HIV acquisition risk per receptive and insertive anal intercourse with HIV infected partner.

Model Equations

Parameters

In general:

- Risk status {1 (low risk), 2 (high risk)}
- Role status $j \in$ {1 (insertive), 2 (receptive), 3 (versatile)}
- Age status $k \in$ {1 (young), 2 (middle), 3 (old)}
- Prevention status $q \in$ { O (no biomedical prevention coverage), P (PrEP), V (vaccine), PV (PrEP and vaccine)}
- Disease stage $\phi \in$ {1 (acute), 2 (CD4>500), 3 (CD4 350-500), 4 (CD4 200-350), 5 (CD4<200)}
- Awareness status $A \in$ { I (Infected), P (Infected on PrEP), D (Diagnosed), E (Engaged in care), U (On ART, Unsuppressed), T (On ART, Suppressed)}

ν^i : Fraction of new PrEP from susceptibles OFF PrEP with risk status i

v_q : Fraction of uninfected population vaccinated during 5-year campaigns starting 2025 by PrEP status q

ω_q : Fraction of discontinuing PrEP from susceptibles ON PrEP with risk status i and prevention status

d_k : Death rate (non-HIV related) for age

μ_ϕ: HIV-related death rate for disease stage

a_k : Aging rate from age k to age

$\rho_{i,k}$: Fraction of population with risk status i and in age group

r_j : Fraction of population with role status

b : Population birth (aging into population) rate

$\sigma_{A,\phi}$: Forward awareness transfer rate based on disease stage ϕ and awareness status A (vertical flows)

$\tau_{A,\phi}$: Drop rate from awareness status A (losing suppression, dropping ART or leaving care) into disease stage

ϵ_{Φ}^{A}: Disease progression rate based on disease stage ϕ and awareness/treatment status A (horizontal flows)

$\lambda^{i,j,k}$: Force of infection for newly infected population entering

$\lambda_P^{i,j,k}$: Force of infection for newly infected population using PrEP entering

$\lambda_V^{i,j,k}$: Force of infection for newly infected vaccinated population entering $I_1^{i,j,k}$

$\lambda_{PV}^{i,j,k}$: Force of infection for newly infected vaccinated population using PrEP entering

$S_q^{i,j,k}$: Susceptible population with prevention status q, risk status i, role group j and age group

$I_\phi^{i,j,k}$: Infected population OFF PrEP with risk status i, role group j and age group k who are in disease stage

$I_{P,\phi}^{i,j,k}$: Infected population ON PrEP with risk status i, role group j and age group k who are in disease stage

$D_\phi^{i,j,k}$: Diagnosed infected population with risk status i, role group j and age group k who are in disease stage

$E_\phi^{i,j,k}$: Infected population who are engaged in care but not on ART with risk status i, role group j and age group k who are in disease stage

$U_\phi^{i,j,k}$: Infected population who on ART but unsuppressed with risk status i, role group j and age group k who are in disease stage

$T_\phi^{i,j,k}$: Infected population who on ART and suppressed with risk status i, role group j and age group k who are in disease stage

I : Infected population size $I = \sum_{i,j,k,\phi}\left(I_\phi^{i,j,k} + I_{P,\phi}^{i,j,k} + D_\phi^{i,j,k} + E_\phi^{i,j,k} + U_\phi^{i,j,k} + T_\phi^{i,j,k}\right)$. Here

S : Susceptible population size $S = \sum_{i,j,k,q}\left(S_q^{i,j,k}\right)$

N : Total population size $N = S + I$

$\delta_{i,j}$: Kronecker delta function

Equations

For simplicity, all variables corresponding to age group have a value 0.

Between vaccination campaigns:

$$\frac{dS^{i,j,k}}{dt} = b\delta_{1,i}r_j\rho_{i,k}N + \omega_P S_P^{i,j,k} + a_{k-1}\frac{\rho_{i,k}}{\sum_l \rho_{l,k}}\sum_l S^{l,j,k-1} - (d_k + \nu^i + a_k)S^{i,j,k} - \lambda^{i,j,k}S^{i,j,k},$$

$$\frac{dS_P^{i,j,k}}{dt} = \nu^i\ S^{i,j,k} + a_{k-1}\frac{\rho_{i,k}}{\sum_l \rho_{l,k}}\sum_l S_P^{l,j,k-1} - \left(d_k + \omega_P\ + a_k\right)S_P^{i,j,k} - \lambda_P^{i,j,k}S_P^{i,j,k},$$

$$\frac{dS_V^{i,j,k}}{dt} = \omega_{PV}S_{PV}^{i,j,k} + a_{k-1}\frac{\rho_{i,k}}{\sum_l \rho_{l,k}}\sum_l S_V^{l,j,k-1} - (d_k + a_k)S_V^{i,j,k} - \lambda_V^{i,j,k}S_V^{i,j,k},$$

$$\frac{dS_{PV}^{i,j,k}}{dt} = a_{k-1}\frac{\rho_{i,k}}{\sum_l \rho_{l,k}}\sum_l S_{PV}^{l,j,k-1} - \left(d_k + \omega_{PV} + a_k\right)S_{PV}^{i,j,k} - \lambda_{PV}^{i,j,k}S_{PV}^{i,j,k},$$

$$\frac{dI_1^{i,j,k}}{dt} = \lambda^{i,j,k}S^{i,j,k} + \lambda_V^{i,j,k}S_V^{i,j,k} + a_{k-1}\frac{\rho_{i,k}}{\sum_l \rho_{l,k}}\sum_l I_1^{l,j,k-1} - (d_k + \epsilon_{I,1} + a_k)I_1^{i,j,k},$$

$$\frac{dI_{\phi}^{i,j,k}}{dt} = \epsilon_{I,\phi-1} I_{\phi-1}^{i,j,k} + a_{k-1}\frac{\rho_{i,k}}{\sum_l \rho_{l,k}}\sum_l I_{\phi}^{l,j,k-1} - (\mu_\phi + d_k + \epsilon_{I,\phi} + \sigma_{I,\phi} + a_k) I_{\phi}^{i,j,k}, \quad \phi = 2, \dots, 5$$

$$\frac{dI_{P,1}^{i,j,k}}{dt} = \lambda_P^{i,j,k} S_P^{i,j,k} + \lambda_{PV}^{i,j,k} S_{PV}^{i,j,k} + a_{k-1}\frac{\rho_{i,k}}{\sum_l \rho_{l,k}}\sum_l I_{P,1}^{l,j,k-1} - (d_k + \epsilon_{P,1} + \sigma_{P,1} + a_k) I_{P,1}^{i,j,k},$$

$$\frac{dI_{P,\phi}^{i,j,k}}{dt} = \epsilon_{P,\phi-1} I_{P,\phi-1}^{i,j,k} + a_{k-1}\frac{\rho_{i,k}}{\sum_l \rho_{l,k}}\sum_l I_{P,\phi}^{l,j,k-1} - (\mu_\phi + d_k + \epsilon_{P,\phi} + \sigma_{P,\phi} + a_k) I_{P,\phi}^{i,j,k}, \quad \phi = 2, \dots, 5$$

$$\frac{dD_1^{i,j,k}}{dt} = \sigma_{P,1} I_{P,1}^{i,j,k} + a_{k-1}\frac{\rho_{i,k}}{\sum_l \rho_{l,k}}\sum_l D_1^{l,j,k-1} - (d_k + \epsilon_{D,1} + \sigma_{D,1} + a_k) D_1^{i,j,k},$$

$$\frac{dD_{\phi}^{i,j,k}}{dt} = \epsilon_{D,\phi-1} D_{\phi-1}^{i,j,k} + \sigma_{P,\phi} I_{P,\phi}^{i,j,k} + \sigma_{I,\phi} I_{\phi}^{i,j,k} + \tau_{E,\phi} E_{\phi}^{i,j,k} + a_{k-1}\frac{\rho_{i,k}}{\sum_l \rho_{l,k}}\sum_l D_{\phi}^{l,j,k-1} - (\mu_\phi + d_k + \epsilon_{D,\phi} + \sigma_{D,\phi} + a_k) D_{\phi}^{i,j,k}, \quad \phi = 2, \dots, 5$$

$$\frac{dE_1^{i,j,k}}{dt} = \sigma_{D,1} D_1^{i,j,k} + a_{k-1}\frac{\rho_{i,k}}{\sum_l \rho_{l,k}}\sum_l E_1^{l,j,k-1} - (d_k + \epsilon_{E,1} + \sigma_{E,1} + a_k) E_1^{i,j,k},$$

$$\frac{dE_{\phi}^{i,j,k}}{dt} = \epsilon_{E,\phi-1} E_{\phi-1}^{i,j,k} + \sigma_{D,\phi} D_{\phi}^{i,j,k} + \tau_{U,\phi} U^{i,j,k} + a_{k-1}\frac{\rho_{i,k}}{\sum_l \rho_{l,k}}\sum_l E_{\phi}^{l,j,k-1} - (\mu_\phi + d_k + \tau_{E,\phi} + \epsilon_{E,\phi} + \sigma_{E,\phi} + a_k) E_{\phi}^{i,j,k}, \quad \phi = 2, \dots, 5$$

$$\frac{dU_1^{i,j,k}}{dt} = \sigma_{E,1} E_1^{i,j,k} + a_{k-1}\frac{\rho_{i,k}}{\sum_l \rho_{l,k}}\sum_l U_1^{l,j,k-1} - (d_k + \epsilon_{U,1} + \sigma_{U,1} + a_k) U_1^{i,j,k},$$

$$\frac{dU_{\phi}^{i,j,k}}{dt} = \epsilon_{U,\phi-1} U_{\phi-1}^{i,j,k} + \sigma_{E,\phi} E_{\phi}^{i,j,k} + \tau_{T,\phi} T_{\phi}^{i,j,k} + a_{k-1}\frac{\rho_{i,k}}{\sum_l \rho_{l,k}}\sum_l U_{\phi}^{l,j,k-1} - (\mu_\phi + d_k + \tau_{U,\phi} + \epsilon_{U,\phi} + \sigma_{U,\phi} + a_k) U_{\phi}^{i,j,k}, \quad \phi = 2, \dots, 5$$

$$\frac{dT_2^{i,j,k}}{dt} = \sigma_{U,1} U_1^{i,j,k} + \sigma_{U,2} U_2^{i,j,k} + a_{k-1}\frac{\rho_{i,k}}{\sum_l \rho_{l,k}}\sum_l T_2^{l,j,k-1} - (d_k + \tau_{T,\phi} + a_k) T_2^{i,j,k},$$

$$\frac{dT_{\phi}^{i,j,k}}{dt} = \sigma_{U,\phi} U_{\phi}^{i,j,k} + a_{k-1}\frac{\rho_{i,k}}{\sum_l \rho_{l,k}}\sum_l T_{\phi}^{l,j,k-1} - (d_k + \tau_{T,\phi} + a_k) T_{\phi}^{i,j,k}, \quad \phi = 3,4,5$$

At the time of vaccination campaigns:

$$S_0^{i,j,k} = (1 - v_0)(S_0^{i,j,k} + S_V^{i,j,k})$$
$$S_V^{i,j,k} = v_0 (S_0^{i,j,k} + S_V^{i,j,k})$$
$$S_P^{i,j,k} = (1 - v_p)(S_P^{i,j,k} + S_{PV}^{i,j,k})$$
$$S_{PV}^{i,j,k} = v_p (S_P^{i,j,k} + S_{PV}^{i,j,k})$$

Force of Infection

The number of new infections among the susceptible class $S^{i,j,k}$ (risk status *i*, role status *j* and age group *k*) due to contacts with the infected class $W_{\phi}^{x,y,z}$ (risk status *x*, role status *y* and age group *z* who are in disease stage ϕ) are calculated using the following rule and accounting for the biomedical HIV protection method used (none, PrEP, vaccine or both) :

Number Estimated likelihood that probability that annual

Field Code Changed

susceptible in class $S^{i,j,k}$	X	# partners for age group *k* with risk status *i*	X	the partner is from age group *z* with risk status *x* and role status *y*	X	the partner is infected in the awareness/ treatment state W and disease stage ϕ	X	transmission probability in partnership between $S^{i,j,k}$ and $W_{\phi}^{x,y,z}$

Field Code Changed

Partnership likelihood is based on mixing matrices by age, risk and role, i.e, proportion of partnership that a person from each group has with every other group. These matrices are updated at each step to balance the participating parties (see below). Age/risk mixing is assumed independent from role mixing.

As a result, the force of infection on a susceptible individual who is not vaccinated nor using PrEP is:

$$\lambda^{i,j,k} = \eta^{i,k} \sum_{x,y,z} m_r^{i,k,x,z}\, m_\rho^{j,y} \Big(\sum_\phi \big(1 - (1-(1-\alpha_c)\theta_\phi\beta)^{(1-\psi^{j,y})c_0\, n_\phi n^{i,x}} \cdot (1-\theta_\phi\beta)^{(1-\psi^{j,y})(1-c_0)n_\phi n^{i,x}} \cdot$$

$$(1-(1-\alpha_c)\theta_R\theta_\phi\beta)^{\psi^{j,y}c_0\, n_\phi n^{i,x}} \cdot (1-\theta_R\theta_\phi\beta)^{\psi^{j,y}(1-c_0)n_\phi n^{i,x}}\big) \frac{\left(I_\phi^{x,y,z} + I_{P,\phi}^{x,y,z} + D_\phi^{x,y,z} + E_\phi^{x,y,z}\right)}{N^{x,y,z}} +$$

$$\sum_\phi \big(1 - (1-(1-\alpha_c)\theta_\phi\beta(1-\alpha_{U,art}))^{(1-\psi^{j,y})c_0\, n_\phi n^{i,x}} \cdot (1-\theta_\phi\beta(1-\alpha_{U,art}))^{(1-\psi^{j,y})(1-c_0)n_\phi n^{i,x}} \cdot$$

$$(1-(1-\alpha_c)\theta_R\theta_\phi\beta(1-\alpha_{U,art}))^{\psi^{j,y}c_0\, n_\phi n^{i,x}} \cdot (1-\theta_R\theta_\phi\beta(1-\alpha_{U,art}))^{\psi^{j,y}(1-c_0)n_\phi n^{i,x}}\big) \frac{U_\phi^{x,y,z}}{N^{x,y,z}}\Big).$$

The force of infection on a PrEP user who is not vaccinated is:

$$\lambda_P^{i,j,k} = \eta^{i,k} \sum_{x,y,z} m_r^{i,k,x,z}\, m_\rho^{j,y} \Big(\sum_\phi \big(1 - (1-(1-\alpha_c)\theta_\phi\beta(1-\alpha_p))^{(1-\psi^{j,y})c_P\, n_\phi n^{i,x}} \cdot$$

$$(1-\theta_\phi\beta(1-\alpha_p))^{(1-\psi^{j,y})(1-c_P)n_\phi n^{i,x}} \cdot (1-(1-\alpha_c)\theta_R\theta_\phi\beta(1-\alpha_p))^{\psi^{j,y}c_P\, n_\phi n^{i,x}} \cdot$$

$$(1-\theta_R\theta_\phi\beta(1-\alpha_p))^{\psi^{j,y}(1-c_P)n_\phi n^{i,x}}\big) \frac{\left(I_\phi^{x,y,z} + I_{P,\phi}^{x,y,z} + D_\phi^{x,y,z} + E_\phi^{x,y,z}\right)}{N^{x,y,z}} +$$

$$\sum_\phi \big(1 - (1-(1-\alpha_c)\theta_\phi\beta(1-\alpha_p)(1-\alpha_{U,art}))^{(1-\psi^{j,y})c_P\, n_\phi n^{i,x}} \cdot$$

$$(1-\theta_\phi\beta(1-\alpha_p)(1-\alpha_{U,art}))^{(1-\psi^{j,y})(1-c_P)n_\phi n^{i,x}} \cdot$$

$$(1-(1-\alpha_c)\theta_R\theta_\phi\beta(1-\alpha_p)(1-\alpha_{U,art}))^{\psi^{j,y}c_P\, n_\phi n^{i,x}} \cdot$$

$$(1-\theta_R\theta_\phi\beta(1-\alpha_p)(1-\alpha_{U,art}))^{\psi^{j,y}(1-c_P)n_\phi n^{i,x}}\big) \frac{U_\phi^{x,y,z}}{N^{x,y,z}}\Big).$$

The force of infection on a susceptible individual who is vaccinated but not using PrEP is:

$$\lambda_V^{i,j,k} = \eta^{i,k} \sum_{x,y,z} m_r^{i,k,x,z} m_\rho^{j,y} \Big(\sum_\phi \Big(1-(1-(1-\alpha_c)\theta_\phi\beta(1-\alpha_v))^{(1-\psi^{j,y})c_V n_\phi n^{i,x}} \cdot$$

$$(1-\theta_\phi\beta(1-\alpha_v))^{(1-\psi^{j,y})(1-c_V)n_\phi n^{i,x}} \cdot (1-(1-\alpha_c)\theta_R\theta_\phi\beta(1-\alpha_v))^{\psi^{j,y}c_V n_\phi n^{i,x}} \cdot$$

$$(1-\theta_R\theta_\phi\beta(1-\alpha_v))^{\psi^{j,y}(1-c_V)n_\phi n^{i,x}}\Big) \frac{\left(I_\phi^{x,y,z} + I_{P,\phi}^{x,y,z} + D_\phi^{x,y,z} + E_\phi^{x,y,z}\right)}{N^{x,y,z}} +$$

$$\sum_\phi \Big(1-(1-\alpha_c)\theta_\phi\beta(1-\alpha_v)(1-\alpha_{U,art}))^{(1-\psi^{j,y})c_V n_\phi n^{i,x}} \cdot$$

$$(1-\theta_\phi\beta(1-\alpha_v)(1-\alpha_{U,art}))^{(1-\psi^{j,y})(1-c_V)n_\phi n^{i,x}} \cdot$$

$$(1-(1-\alpha_c)\theta_R\theta_\phi\beta(1-\alpha_v)(1-\alpha_{U,art}))^{\psi^{j,y}c_V n_\phi n^{i,x}} \cdot$$

$$(1-\theta_R\theta_\phi\beta(1-\alpha_v)(1-\alpha_{U,art}))^{\psi^{j,y}(1-c_V)n_\phi n^{i,x}}\Big) \frac{U_\phi^{x,y,z}}{N^{x,y,z}}\Big).$$

The force of infection on an individual who is vaccinated but not using PrEP is:

$$\lambda_{PV}^{i,j,k} = \eta^{i,k} \sum_{x,y,z} m_r^{i,k,x,z}\, m_\rho^{j,y} \Big(\sum_{\phi} (1-(1-(1-\alpha_c)\theta_\phi\beta(1-\alpha_p)(1-\alpha_v))^{(1-\psi^{j,y})c_{PV}n_\phi n^{i,x}} \cdot$$
$$(1-\theta_\phi\beta(1-\alpha_p)(1-\alpha_v))^{(1-\psi^{j,y})(1-c_{PV})n_\phi n^{i,x}} \cdot (1-(1-\alpha_c)\theta_R\theta_\phi\beta(1-\alpha_p)(1-\alpha_v))^{\psi^{j,y}c_{PV}n_\phi n^{i,x}} \cdot$$
$$(1-\theta_R\theta_\phi\beta(1-\alpha_p)(1-\alpha_v))^{\psi^{j,y}(1-c_{PV})n_\phi n^{i,x}}) \frac{(I_\phi^{x,y,z} + I_{P,\phi}^{x,y,z} + D_\phi^{x,y,z} + E_\phi^{x,y,z})}{N^{x,y,z}} +$$
$$\sum_{\phi} (1-(1-\alpha_c)\theta_\phi\beta(1-\alpha_p)(1-\alpha_v)(1-\alpha_{U,art}))^{(1-\psi^{j,y})c_{PV}n_\phi n^{i,x}} \cdot$$
$$(1-\theta_\phi\beta(1-\alpha_p)(1-\alpha_v)(1-\alpha_{U,art}))^{(1-\psi^{j,y})(1-c_{PV})n_\phi n^{i,x}} \cdot$$
$$(1-(1-\alpha_c)\theta_R\theta_\phi\beta(1-\alpha_p)(1-\alpha_v)(1-\alpha_{U,art}))^{\psi^{j,y}c_{PV}n_\phi n^{i,x}} \cdot$$
$$(1-\theta_R\theta_\phi\beta(1-\alpha_p)(1-\alpha_v)(1-\alpha_{U,art}))^{\psi^{j,y}(1-c_{PV})n_\phi n^{i,x}}) \frac{U_\phi^{x,y,z}}{N^{x,y,z}}\Big).$$

Here:

$\eta^{i,k}$: number of partners for individuals in risk group i and age group ,

$n^{i,x}$: number of acts per year in partnership between risk group i and risk group x ,

n_ϕ: relative sexual activity (multiplier for the number of acts per year) in partnership in which the infected partner is in disease stage ϕ to account for reduced sexual activity during late HIV stages using CD4 >500 as a reference,

$m_\rho^{i,k,x,z}$: probability for a partner of a person with risk status i from age group k to be with risk status x from age group z (risk/age mixing),

$m_r^{j,y}$: mixing probability between people with role j and role y (role mixing),

α_c : condom efficacy in reducing susceptibility per act,

c_q : rate of condom use by uninfected individuals with prevention status *q*,

θ_ϕ : relative HIV transmission risk per act by disease stage ϕ , using asymptomatic stage (CD4 >500) as a reference

β : HIV-transmission risk per unprotected insertive anal act from untreated infected MSM in asymptomatic stage (CD4>500) to uninfected MSM not using PrEP or vaccine,

$\psi^{j,y}$: fraction of receptive acts when MSM with role status *j* is partnering with MSM with role status *y*,

θ_R : relative HIV acquisition risk per receptive anal act compared to insertive anal acts,

$\alpha_{U,art}$: ART efficacy in reducing infectiousness per act when virally unsuppressed,

α_p: PrEP efficacy in reducing susceptibility per act,

α_v: Vaccine efficacy in reducing susceptibility per act,

$N^{x,y,z}$: Population size with risk status *x*, role status *y*. and age group *z*

$N^{x,y,z} = \sum_q S_q^{x,y,z} + \sum_\phi \left(I_\phi^{x,y,z} + I_{P,\phi}^{x,y,z} + D_\phi^{x,y,z} + E_\phi^{x,y,z} + F_\phi^{x,y,z} + T_\phi^{x,y,z} \right)$. Here $T_1^{x,y,z} = 0$.

Partnership Balancing

Balancing by age and risk

We consider mixing between all possible 6 age/risk groups. Likelihood of partnerships between 2 age/risk groups are stored in 6x6 age/risk mixing matrix $\boldsymbol{A}=\boldsymbol{a_{ij}}$ representing the likelihood that each partner of an individual from group i to be from group j. $\boldsymbol{A}$ is informed initially by data representative for the MSM population in Seattle. The matrix is constantly updated to balance the number of partnerships between different age/risk groups. The procedure aims to equilize the number of people participating at each end of the partnerships between 2 different age/risk groups (i and j) calculated as:

(Number of people in group i)*(Number of partners per individual)*(Likelihood a_{ij})

At each time step, the balancing procedure proceeds as follows:

1) All off-diagonal pairs a_{ij} and a_{ij} are adjusted to equilize the number of people participating at each end of the partnerships. Adjusted is the entry which leads to decrease in the sum of the off-diagonal values
2) Diagonal entries are adjusted to guarantee that each row sums up to 1.

Step 1) of the procedure guarantees that the sum of the off-diagonal entries decreases and therefore remains below 1. This makes step 2) always possible. The procedure favors sexual mixing within each age/risk group which is supported by self-reported behavioral data.

Table S2. Initial value of the mixing matrix used in the simulations

Mixing likelihoods	Young, low risk	Middle, low risk	Old, low risk	Young, high risk	Middle, high risk	Old, high risk
Young, low risk	0.2394	0.0986	0.1925	0.2066	0.0751	0.1878
Middle, low risk	0.1323	0.254	0.2222	0.0899	0.127	0.1746
Old, low risk	0.1666	0	0.381	0.1111	0	0.3413
Young, high risk	0.1231	0.0313	0.0901	0.3217	0.1176	0.3162
Middle, high risk	0.0899	0.1013	0.0727	0.1396	0.3327	0.2638
Old, high risk	0.0806	0	0.1573	0.2258	0	0.5363

Balancing by role

Fractions of partners by role are constantly updated to balance the number of partnerships between different role groups.

We use the following likelihood matrix of partnering between sexual role groups:

Table S3. Sexual partnership mixing matrix

Role group	Receptive	Insertive	Versatile
Receptive	a	b	1-a-b
Insertive	x	c	1-c-x
Versatile	y	z	1-y-z

Assuming that the overall number of partners per year is the same for each role group our balancing procedure requires that:
$x*N_{ins}= b*N_{rec}$
$y*N_{ver}= (1-a-b) *N_{rec}$
$z*N_{ver}= (1-c-x) *N_{ins}$

In all simulations, the values of a, b and c remain constant while x, y and z are updated at each time step. Simulations which result in negative mixing rates are discarded.

Model Inputs

Table S4. Complete list of fixed parameter values used in the analysis

Variable name	Description	Value
N_0	Initial population size in 2004	45000
	Initial fraction of infected population who are on ART	0.83
	Initial fraction of the infected population on ART who are virally suppressed	0.83
ν^1	Fraction of low risk population starting PrEP annually after 2014	0.06
ν^2	Fraction of high risk population starting PrEP annually after 2014	0.25
v_0	Fraction of uninfected population vaccinated during 5-year campaigns starting 2025	0.6
v_P	Fraction of PrEP users vaccinated during 5-year campaigns starting 2025	0.9
ω_P	Annual rate of dropping PrEP if unvaccinated	0.2
ω_{PV}	Annual rate of dropping PrEP if vaccinated	1
$1/a_1$	Duration of the young age group (15-24)	10 years
$1/a_2$	Duration of the middle age age group (25-44)	20 years
$1/a_3$	Duration of the old age group (45-64)	20 years
used in $\rho_{i,1}$	Fraction of the population in the young age group	0.168
used in $\rho_{i,2}$	Fraction of the population in the middle age group	0.463
used in $\rho_{i,1}$	Fraction of MSM in the young age group with high risk status	0.31
used in $\rho_{i,2}$	Fraction of MSM in the middle age group with high risk status	0.099
used in $\rho_{i,3}$	Fraction of MSM in the old age group with high risk status	0.065

d_1	Death rate (non-HIV related) for the young age group	0.001319
d_2	Death rate (non-HIV related) for the middle age group	0.001574
d_3	Death rate (non-HIV related) for the oldage group	0.008438
used in r_1	Fraction of MSM with insertive role status	0.3248
used in r_2	Fraction of MSM with versatile role group	0.2683
b	Population recruitment rate (MSM turning 15)	0.03
$\sigma_{P,\phi}$	Annual rate of HIV screening for PrEP users	2
$\sigma_{I,1}$	Annual rate of HIV diagnosis for acutely infected MSM, unvaccinated, not using PrEP	0
$\sigma_{I,2\text{-}4}$	Rate of HIV diagnosis for infected MSM with CD4 > 200	Calculated to match overall rate
$1/\epsilon_{A,1}$, A in {I, P}	Duration of acute stage	0.33 years
$1/\epsilon_{A,2}$, A in {I,P,D,E,U}	Duration of HIV stage with CD4>500	3.32 years
$1/\epsilon_{A,3}$, A in {I,P,D,E,U}	Duration of HIV stage with CD4 350-500	2.7 years
$1/\epsilon_{A,4}$, A in {I,P,D,E,U}	Duration of HIV stage with CD4 200-350	5.5 years
$1/\epsilon_{A,5}$, A in {I,P,D,E,U}	Duration of HIV stage with CD4<200	5.06 years
μ_2	HIV-related death rate for MSM with CD4>500, off ART	0.0032
μ_3	HIV-related death rate for MSM with CD4 350-500, off ART	0.0039
μ_4	HIV-related death rate for MSM with CD4 200-350, off ART	0.009
$\eta^{2,1}$	Annual number of partners for MSM in the young age group with high risk status	10.5
$\eta^{2,2}$	Annual number of partners for MSM in the middle age group with high risk status	10.5
$\eta^{2,3}$	Annual number of partners for MSM in the old age group with high risk status	6
$\eta^{1,1}$	Annual number of partners for MSM in the young age group with low risk status	1.5
$\eta^{1,2}$	Annual number of partners for MSM in the middle age group with low risk status	1.5
$\eta^{1,3}$	Annual number of partners for MSM in the old age group with low risk status	1
θ_5	Multiplier for reduced sexual activity when partner is in AIDS stage (CD4<200)	0.75
$m^{1,1}$	Probability for someone in role group "insertive" to partner with someone in role group "insertive"	0.18
$m^{1,3}$	Probability for someone in role group "receptive" to partner with someone in role group "insertive"	0.6
$m^{3,3}$	Probability for someone in role group "receptive" to partner with someone in role group "receptive"	0.08
c_0 and c_V	Fraction of acts which are protected by a condom when PrEP is not used	0.63
c_P and c_{PV}	Fraction of acts of PrEP users, protected by a condom	0.125
θ_1	Relative infectiousness of acutely infected MSM compared to MSM with CD4>200	26

θ_5	Relative infectiousness of MSM with CD4<200 compared to MSM with CD4>200	3
$\psi^{1,1}$	Fraction of receptive acts in partnership between two MSM with insertive role status	0.5
$\psi^{1,2}$	Fraction of receptive acts when MSM with insertive role status is partnering with MSM with receptive role status	0
$\psi^{1,3}$	Fraction of receptive acts when MSM with insertive role status is partnering with MSM with versatile role status	0
$\psi^{2,1}$	Fraction of receptive acts when MSM with receptive role status is partnering with MSM with insertive role status	1
$\psi^{2,2}$	Fraction of receptive acts in partnership between two MSM with receptive role status	0.5
$\psi^{2,3}$	Fraction of receptive acts when MSM with receptive role status is partnering with MSM with versatile role status	1
$\psi^{3,1}$	Fraction of receptive acts when MSM with versatile role status is partnering with MSM with insertive role status	1
$\psi^{3,2}$	Fraction of receptive acts for MSM with versatile role status partnering MSM with receptive role status	0
$\psi^{3,3}$	Fraction of receptive acts in partnership between two MSM with versatile role status	0.5
	ART efficacy in reducing infectiousness per act when virally suppressed	1
α_p	PrEP efficacy in reducing susceptibility per act	0.8
α_v	Vaccine efficacy in reducing susceptibility per act	0.5

Model Calibration

The model simulations start in 2004, parameterized with values listed in Table 1 and Table S4. We calibrated the model outcomes to match data from 2012 to select parameter sets that best captures the epidemic trends and clinical disease progression among MSM in King County, WA.[36,70] We fit model outcomes to the HIV prevalence and the treatment cascade (% diagnosed, % engaged in care, % on ART, % virally suppressed). Monte Carlo filtering was used to select 100 parameter sets for which all targets are within calibrated ranges listed in Table 1 of the main text. The procedure of parameter sets selection consist of the following:

1) All uncertain parameters are sampled from their ranges in Table S5 and HIV prevalence target is checked against its 2012 target range
2) If HIV prevalence is accepted, then all awareness/treatment parameters (#1 - #12 and #21-#25 in Table S5) are resampled while keeping behavioral and transmission parameters fixed (#13 - #20 in Table S5). This step is repeated up to 50 times while all HIV prevalence and treatment cascade outcomes are within targeted ranges.
3) The procedure is repeated while 100 parameter sets are selected

Table S5. List of parameters ranges used in model calibration

#	Variable name	Description	Range
1	$\sigma_{I,5}$	Annual rate of HIV diagnosis for infected MSM with CD4<200 in absence of intervention	0.2-0.4
2		Overall rate of HIV diagnosis for infected MSM annually	0.2-0.3
3	$\sigma_{D,2\text{-}4}$	Annual rate of engagement in care for acutely diagnosed MSM with CD4>200	0.5-1
4	$\sigma_{D,5}$	Annual rate of engagement in care for acutely diagnosed MSM with CD4<200	0.5-1
5	$\sigma_{E,2\text{-}5}$	Annual rate of ART initiation for MSM in care	0.5-1
6	$\sigma_{U,2\text{-}4}$	Annual rate of achieving viral suppression on ART for MSM with CD4>200	2.0-6.0
7	$\sigma_{U,5}$	Annual rate of achieving viral suppression on ART for MSM with CD4<200	1.0-3.0
8	$\tau_{E,2\text{-}4}$	Rate at which engaged in care MSM with CD4>200 drop from care	0.2-0.3
9	$\tau_{E,5}$	Rate at which engaged in care MSM with CD4<200 drop from care	0.1-0.2
10	$\tau_{U,2\text{-}5}$	Annual ART drop rate	0.2-0.25
11	$\tau_{T,2\text{-}5}$	Rate of losing virally suppression due to inconsistent use of ART	0.2-0.3
12	used in $\epsilon_{U,i}$	Multiplicative factor which extends duration of HIV stages while on ART with detectible viral load (virally unsuppressed)	1-1.5
13	used in $\eta^{2,k}$	Multiplicative factor which adjusts for overreported (overlapping) partnerships of high-risk MSM	0.5-1
14	used in $\eta^{1,k}$	Multiplicative factor which adjusts for overreported (overlapping) partnerships of low-risk MSM	0.78-1
15	$n^{1,1}$	Number of acts per year in partnership in which both MSM have low risk status	40-60
16	$n^{1,2}$	Number of acts per year in partnership between MSM with low risk status	2.0-5.0
17	$n^{2,2}$	Number of acts per year in partnership in which both MSM have high risk status	1.0-2.0
18	α_c	Condom efficacy in reducing infectiousness per act	0.7-0.9
19	β	HIV-transmission probability per unprotected insertive anal act with untreated infected MSM with CD4>200	0.001-0.002
20	θ_R	Relative HIV acquisition risk per receptive sex act compared to insertive act	3.0-7.0
21	$\boldsymbol{\alpha_{U,art}}$	ART efficacy in reducing infectiousness per act when virally unsuppressed	0.3-0.7
22		HIV prevalence in 2004	0.14-0.16
23		Initial fraction of infected MSM (CD4>200) who are diagnosed	0.8-0.9
24		Initial fraction of infected MSM (CD4<200) who are diagnosed	0.94-0.98
25		Initial fraction of diagnosed MSM who are engaged in care	0.8-0.9

Given the starting population size of 45,000 MSM in 2004, the model estimates growth to 62,388 MSM by 2045.

Table S6. Description of health states and corresponding utility weight

Health State	HIV status	Aware Status	Stage of Care	cd4cat	CD4	Risk Level	ART	Utility Weight
SL	susceptible	S	Susceptible	NA	NA	NA	NA	1.00
SH	susceptible	S	Susceptible	NA	NA	NA	NA	1.00
SPL	susceptible	S	Susceptible	NA	NA	NA	NA	1.00
SPH	susceptible	S	Susceptible	NA	NA	NA	NA	1.00
SVL	susceptible	S	Susceptible	NA	NA	NA	NA	1.00
SVH	susceptible	S	Susceptible	NA	NA	NA	NA	1.00
SPVL	susceptible	S	Susceptible	NA	NA	NA	NA	1.00
SPVH	susceptible	S	Susceptible	NA	NA	NA	NA	1.00
I1L	infected	I	Infected and unaware	1	Acute	low	0	0.69
I1H	infected	I	Infected and unaware	1	Acute	high	0	0.69
I2L	infected	I	Infected and unaware	2	>500	low	0	0.73
I2H	infected	I	Infected and unaware	2	>500	high	0	0.73
I3L	infected	I	Infected and unaware	3	350-500	low	0	0.71
I3H	infected	I	Infected and unaware	3	350-500	high	0	0.71
I4L	infected	I	Infected and unaware	4	200-349	low	0	0.69
I4H	infected	I	Infected and unaware	4	200-349	high	0	0.69
I5L	infected	I	Infected and unaware	5	<200	low	0	0.69
I5H	infected	I	Infected and unaware	5	<200	high	0	0.69
D1L	infected	D	Diagnosed	1	Acute	low	0	0.69

Health State	HIV status	Aware Status	Stage of Care	cd4cat	CD4	Risk Level	ART	Utility Weight
D1H	infected	D	Diagnosed	1	Acute	high	0	0.69
D2L	infected	D	Diagnosed	2	>500	low	0	0.73
D2H	infected	D	Diagnosed	2	>500	high	0	0.73
D3L	infected	D	Diagnosed	3	350-500	low	0	0.71
D3H	infected	D	Diagnosed	3	350-500	high	0	0.71
D4L	infected	D	Diagnosed	4	200-349	low	0	0.69
D4H	infected	D	Diagnosed	4	200-349	high	0	0.69
D5L	infected	D	Diagnosed	5	<200	low	0	0.69
D5H	infected	D	Diagnosed	5	<200	high	0	0.69
E1L	infected	E	Enaged (not on ART)	1	Acute	low	0	0.69
E1H	infected	E	Enaged (not on ART)	1	Acute	high	0	0.69
E2L	infected	E	Enaged (not on ART)	2	>500	low	0	0.73
E2H	infected	E	Enaged (not on ART)	2	>500	high	0	0.73
E3L	infected	E	Enaged (not on ART)	3	350-500	low	0	0.71
E3H	infected	E	Enaged (not on ART)	3	350-500	high	0	0.71
E4L	infected	E	Enaged (not on ART)	4	200-349	low	0	0.69
E4H	infected	E	Enaged (not on ART)	4	200-349	high	0	0.69
E5L	infected	E	Enaged (not on ART)	5	<200	low	0	0.69
E5H	infected	E	Enaged (not on ART)	5	<200	high	0	0.69
U1L	infected	U	Unsuppressed and ON ART	1	Acute	low	1	0.69
U1H	infected	U	Unsuppressed and ON ART	1	Acute	high	1	0.69
U2L	infected	U	Unsuppressed and ON ART	2	>500	low	1	0.73
U2H	infected	U	Unsuppressed and ON ART	2	>500	high	1	0.73
U3L	infected	U	Unsuppressed and ON ART	3	350-500	low	1	0.71

Health State	HIV status	Aware Status	Stage of Care	cd4cat	CD4	Risk Level	ART	Utility Weight
U3H	infected	U	Unsuppressed and ON ART	3	350-500	high	1	0.71
U4L	infected	U	Unsuppressed and ON ART	4	200-349	low	1	0.69
U4H	infected	U	Unsuppressed and ON ART	4	200-349	high	1	0.69
U5L	infected	U	Unsuppressed and ON ART	5	<200	low	1	0.69
U5H	infected	U	Unsuppressed and ON ART	5	<200	high	1	0.69
T1L	infected	T	Suppressed	1	Acute	low	1	0.73
T1H	infected	T	Suppressed	1	Acute	high	1	0.73
T2L	infected	T	Suppressed	2	>500	low	1	0.73
T2H	infected	T	Suppressed	2	>500	high	1	0.73
T3L	infected	T	Suppressed	3	350-500	low	1	0.73
T3H	infected	T	Suppressed	3	350-500	high	1	0.73
T4L	infected	T	Suppressed	4	200-349	low	1	0.73
T4H	infected	T	Suppressed	4	200-349	high	1	0.73
T5L	infected	T	Suppressed	5	<200	low	1	0.73
T5H	infected	T	Suppressed	5	<200	high	1	0.73

Table S7. Modeled population sizes at the start and end of the time horizon

Case	Start 2004	Year 2025	End 2045	Growth (no.)	Percent Growth
Reference: PrEP alone	45,000	51606	62388	17389	0.3864
Intervention: Vaccine + PrEP	45,000	51606	62532	17532	0.3896

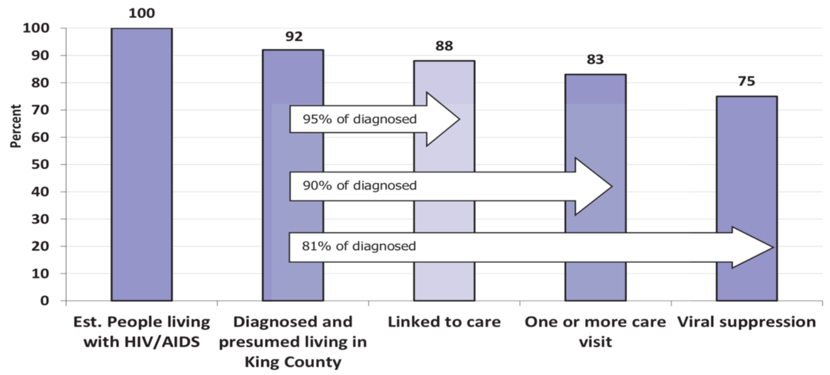

	Estimated people living with HIV/AIDS[a]	Diagnosed and presumed living in King County[b]	Linked to care in 2015[c]	One or more care visit[d]	Viral suppression[e]
Number of people	7400	6808	228/239	6157	5524

Figure S2. King County HIV Care Continuum in 2015

Source: Seattle Public Health King County, (data reported through of June 30, 2016)

[a] Percent undiagnosed was calculated as 6% among MSM for King County; prior estimate of 15% was used for non-MSM (based on CDC and Washington State estimates) resulting in an estimate of 7.3% overall, rounded up to 8% for a slightly more conservative estimate (this may be the most uncertain bar in the continuum). Estimated people living with HIV/AIDS is calculated by dividing "diagnosed and presumed living in King County" residents by 0.92.

[b] Diagnosed cases are those presumed living in King County during 2015. Individuals with no contact for ten or more years were presumed to have relocated or died (N=249). Others with unconfirmed deaths or relocations (identified, for example by online Internet database searches, but not confirmed by the new jurisdiction or another secondary source) and no laboratory results reported for >18 months were also excluded (N=161).

[c] Linked to care in 2015 is not a subset of earlier data (hence different color in the graph) and is based on the percent diagnosed in 2015 with a CD4 or viral load test within 3 months of diagnosis. The percent linked in the figure, 88%, is the percent of diagnosed cases in 2015 who linked (95.4%) times 92.0% to account for undiagnosed cases.

[d] One or more care visit was based on one or more reported laboratory result (CD4, viral load, genotype).

[e] Viral suppression is defined as the most recent viral load test result in 2015 less than 200 copies.

Estimating Cost-Effectiveness

We evaluate ICERs using a cost-effectiveness threshold ranging from 1-3x the GDP per capita, or approximately \$50,000 - \$150,000 per QALY gained using the equation

$$ICER = \frac{Cost_{intervention} - Cost_{standardcare}}{QALYs_{intervention} - QALYs_{standardcare}}.$$

Approach to Health Outcomes

Health impact is measured in HIV infections averted and total QALYs gained, both discounted 3% annually.[62,88] The estimation of compartment size mid-point moments each quarter represent units of one-

quarter person-year time. This "life year" matrix integrates with a function to adjust for health-state specific utility weights and discount 3% annually. The sum of the resulting "QALY matrix" from 2025-2045 represents the total population-level discounted QALYs for a simulation. To calculate average QALYs per person in a model with fluctuating population size, we sum the number of people alive in each year of the model and divide by the time horizon length to find the average population size for the simulation. In summary, person-time in each health state multiplied by the corresponding preference-based utility weights will be summed and discounted 3% annually to estimate total QALYs.[53–57]

Sensitivity Analysis

Table S8. Scenario with 70% vaccine efficacy

Outcome	Reference	Vaccine	Incremental
HIV BURDEN —————-			
New Infections 2025-2045	3,074	1,380	-1,694
Diagnoses 2025-2045	2,935	1,672	-1,263
PLWH 2045	4,806	3,557	-1,249
HIV Prevalence (%) 2045	7.70%	5.68%	-2.02%
UTILIZATION —————			
PrEP Users in 2025	11,233	11,233	0
Total Protected in 2045	14,905	36,987	22,081
—PrEP alone	14,905	5,523	-9,383
—HIV vaccine alone	0	31,435	31,435
—PrEP and HIV vaccine	0	29	29
OUTCOMES ——————			
Life Years, total	1,100,665	1,103,765	3,100
QALYs, total	923,770	924,620	851
COSTS ———————			
Total Cost, $	$2,396,128,051	$2,399,646,724	$3,518,673
—PrEP cost, $	$675,392,497	$225,458,175	-$449,934,322
—Vaccine cost, $	$0	$534,016,765	$534,016,765
—HIV Care Cost, $	$1,720,735,553	$1,640,171,783	-$80,563,770
ICER, $/QALY			$4,136

Table S9. Scenario with 30% vaccine efficacy

Outcome	Reference	Vaccine	Incremental
HIV BURDEN ————-			
New Infections 2025-2045	3,074	2,517	-556
Diagnoses 2025-2045	2,935	2,630	-305
PLWH 2045	4,806	4,408	-398
HIV Prevalence (%) 2045	7.70%	7.06%	-0.65%
UTILIZATION ————			
PrEP Users in 2025	11,233	11,233	0
Total Protected in 2045	14,905	36,322	21,417
—PrEP alone	14,905	5,461	-9,445
—HIV vaccine alone	0	30,832	30,832
—PrEP and HIV vaccine	0	29	29
OUTCOMES —————			
Life Years, total	1,100,665	1,101,670	1,005
QALYs, total	923,770	924,315	546
COSTS ——————			
Total Cost, $	$2,396,128,051	$2,455,514,569	$59,386,518
—PrEP cost, $	$675,392,497	$224,315,801	-$451,076,697
—Vaccine cost, $	$0	$530,444,101	$530,444,101
—HIV Care Cost, $	$1,720,735,553	$1,700,754,667	-$19,980,886
ICER, $/QALY			$108,824

Table S10. Scenario with no condom displacement

Outcome	Reference	Vaccine	Incremental
HIV BURDEN ——————-			
New Infections 2025-2045	3,074	1,827	-1,347
Diagnoses 2025-2045	2,935	2,030	-983
PLWH 2045	4,806	3,842	-964
HIV Prevalence (%) 2045	7.70%	6.14%	-1.56%
UTILIZATION ——————			
PrEP Users in 2025	11,233	11,273	0
Total Protected in 2045	14,905	36,770	21,865
—PrEP alone	14,905	5,506	-9,399
—HIV vaccine alone	0	31,234	31,234
—PrEP and HIV vaccine	0	29	29
OUTCOMES ———————			
Life Years, total	1,100,665	1,103,521	2,857
QALYs, total	923,770	924,335	566
COSTS ————————			
Total Cost, $	$2,396,128,051	$2,408,263,232	$12,135,182
—PrEP cost, $	$675,392,497	$225,534,454	-$449,858,043
—Vaccine cost, $	$0	$533,545,002	$533,545,002
—HIV Care Cost, $	$1,720,735,553	$1,649,183,776	-$71,551,777
ICER, $/QALY			$21,457

Table S11. Scenario with half priced PrEP

Outcome	Reference	Vaccine	Incremental
HIV BURDEN ——————-			
New Infections 2025-2045	3,074	1,910	-1,164
Diagnoses 2025-2045	2,935	2,121	-814
PLWH 2045	4,806	3,949	-857
HIV Prevalence (%) 2045	7.70%	6.31%	-1.39%
UTILIZATION ——————			
PrEP Users in 2025	11,233	11,233	0
Total Protected in 2045	14,905	36,680	21,775
—PrEP alone	14,905	5,494	-9,412
—HIV vaccine alone	0	31,158	31,158
—PrEP and HIV vaccine	0	29	29
OUTCOMES ———————			
Life Years, total	1,100,665	1,102,750	2,086
QALYs, total	923,770	924,486	717
COSTS ————————			
Total Cost, $	$2,080,009,965	$2,321,298,010	$241,288,044
—PrEP cost, $	$359,274,412	$119,641,363	-$239,633,049
—Vaccine cost, $	$0	$532,299,370	$532,299,370
—HIV Care Cost, $	$1,720,735,553	$1,669,357,277	-$51,378,276
ICER, $/QALY			$336,671

Table S12. Scenario with half priced HIV vaccine

Outcome	Reference	Vaccine	Incremental
HIV BURDEN —————-			
New Infections 2025-2045	3,074	1,910	-1,164
Diagnoses 2025-2045	2,935	2,121	-814
PLWH 2045	4,806	3,949	-857
HIV Prevalence (%) 2045	7.70%	6.31%	-1.39%
UTILIZATION —————			
PrEP Users in 2025	11,233	11,233	0
Total Protected in 2045	14,905	36,680	21,775
—PrEP alone	14,905	5,494	-9,412
—HIV vaccine alone	0	31,158	31,158
—PrEP and HIV vaccine	0	29	29
OUTCOMES ——————			
Life Years, total	1,100,665	1,102,750	2,086
QALYs, total	923,770	924,486	717
COSTS ———————			
Total Cost, $	$2,396,128,051	$2,215,705,405	-$180,422,645
—PrEP cost, $	$675,392,497	$224,911,311	-$450,481,187
—Vaccine cost, $	$0	$321,436,818	$321,436,818
—HIV Care Cost, $	$1,720,735,553	$1,669,357,277	-$51,378,276
ICER, $/QALY			-$251,745

Table S13. Scenario with PrEP price doubled

Outcome	Reference	Vaccine	Incremental
HIV BURDEN ——————-			
New Infections 2025-2045	3,074	1,910	-1,164
Diagnoses 2025-2045	2,935	2,121	-814
PLWH 2045	4,806	3,949	-857
HIV Prevalence (%) 2045	7.70%	6.31%	-1.39%
UTILIZATION ——————			
PrEP Users in 2025	11,233	11,233	0
Total Protected in 2045	14,905	36,680	21,775
—PrEP alone	14,905	5,494	-9,412
—HIV vaccine alone	0	31,158	31,158
—PrEP and HIV vaccine	0	29	29
OUTCOMES ———————			
Life Years, total	1,100,665	1,102,750	2,086
QALYs, total	923,770	924,486	717
COSTS ————————			
Total Cost, $	$3,028,364,221	$2,637,107,853	-$391,256,368
—PrEP cost, $	$1,307,628,668	$435,451,206	-$872,177,462
—Vaccine cost, $	$0	$532,299,370	$532,299,370
—HIV Care Cost, $	$1,720,735,553	$1,669,357,277	-$51,378,276
ICER, $/QALY			-$545,922

Table S14. Scenario with HIV vaccine price doubled

Outcome	Reference	Vaccine	Incremental
HIV BURDEN —————-			
New Infections 2025-2045	3,074	1,910	-1,164
Diagnoses 2025-2045	2,935	2,121	-814
PLWH 2045	4,806	3,949	-857
HIV Prevalence (%) 2045	7.70%	6.31%	-1.39%
UTILIZATION —————			
PrEP Users in 2025	11,233	11,233	0
Total Protected in 2045	14,905	36,680	21,775
—PrEP alone	14,905	5,494	-9,412
—HIV vaccine alone	0	31,158	31,158
—PrEP and HIV vaccine	0	29	29
OUTCOMES ——————			
Life Years, total	1,100,665	1,102,750	2,086
QALYs, total	923,770	924,486	717
COSTS ———————			
Total Cost, $	$2,396,128,051	$2,848,293,062	$452,165,011
—PrEP cost, $	$675,392,497	$224,911,311	-$450,481,187
—Vaccine cost, $	$0	$954,024,474	$954,024,474
—HIV Care Cost, $	$1,720,735,553	$1,669,357,277	-$51,378,276
ICER, $/QALY			$630,908

References

1. Emory University Rollins School of Public Health. AIDSVu. www.aidsvu.org.

2. U.S. Census Bureau Population Division. *National Population Projection, 2014*. Washington, DC; 2015.

3. HIV/AIDS Epidemiology Unit, Public Health - Seattle & King County, and Infectious Diseases and Reproductive Health Assessment Unit, Washington State Department of Health. *HIV/AIDS Epidemiology Report*. 2005;65:1-33.

4. Sagaon-teyssier L, Suzan-monti M, Demoulin B, Capitant C, Lorente N. Uptake of PrEP and condom and sexual risk behavior among MSM during the ANRS IPERGAY trial. 2016;28:48-55. doi:10.1080/09540121.2016.1146653

5. Seattle WS, County K, Allen E, et al. EPIDEMIOLOGY REPORT. 2015.

6. Institute for Clinical and Economic Review. *ICER Value Assessment Framework Summary*. Boston; 2015.

7. Gold M, Siegel J, Russell L, Weinstien M. *Cost-Effectiveness in Health and Medicine*. New York, New York: Oxford University Press; 1996.

8. Briggs A, Claxton K, Sculpher M. *Decision Modelling for Health Economic Evaluation*. 1st ed. London: Oxford University Press; 2006.

9. Joyce VR, Barnett PG, Bayoumi AM, et al. Health-related quality of life in a randomized trial of antiretroviral therapy for advanced HIV disease. *Journal of acquired immune deficiency syndromes (1999)*. 2009;50(1):27-36. doi:10.1097/QAI.0b013e31818ce6f3

10. Kauf TL, Roskell N, Shearer A, et al. A predictive model of health state utilities for HIV patients in the modern era of highly active antiretroviral therapy. *Value in health : the journal of the International Society for Pharmacoeconomics and Outcomes Research*. 2008;11(7):1144-1153. doi:10.1111/j.1524-4733.2008.00326.x

11. Bayoumi AM, Barnett PG, Joyce VR, et al. Cost-effectiveness of newer antiretroviral drugs in treatment-experienced patients with multidrug-resistant HIV disease. *Journal of acquired immune deficiency syndromes (1999)*. 2013;64(4):382-391. doi:10.1097/QAI.0000000000000002

12. Sanders GD, Bayoumi AM, Sundaram V, et al. Cost-effectiveness of screening for HIV in the era of highly active antiretroviral therapy. *The New England journal of medicine*. 2005;352(6):570-585. doi:10.1056/NEJMsa042657

13. Tengs TO, Lin TH. A meta-analysis of utility estimates for HIV/AIDS. *Medical decision making : an international journal of the Society for Medical Decision Making*. 2002;22(6):475-481.
