# Supplementary material for: Competing biomedical HIV prevention strategies: potential cost‐effectiveness of HIV vaccines and PrEP in Seattle, WA
Source: J Int AIDS Soc. 2019 Aug 11;22(8):e25373. doi: 10.1002/jia2.25373 (PMC6689690; doi:10.1002/jia2.25373)
Supplement: Supplementary file 3 [file JIA2-22-e25373-s003.docx]

**Appendix S1.** Supplementary material.
